# Subtype‐associated complexity and prognostic significance of the NLRP3 inflammasome landscape in pancreatic neoplasms

**DOI:** 10.1002/2056-4538.70019

**Published:** 2025-02-19

**Authors:** Kristóf Németh, Eszter Mezei, Justína Vörös, Katalin Borka, Adrián Pesti, István Kenessey, András Kiss, András Budai

**Affiliations:** ^1^ Department of Pathology, Forensics and Insurance Medicine Semmelweis University Budapest Hungary; ^2^ National Cancer Registry and Center for Biostatistics National Institute of Oncology Budapest Hungary

**Keywords:** pancreatic neoplasms, IPMN, inflammasomes, interleukin‐18, prognosis, neoplasm invasiveness

## Abstract

Intraductal papillary mucinous neoplasm (IPMN) can progress into malignant pancreatic cancer, posing challenges in accurately assessing the risk of malignancy. While the nucleotide‐binding oligomerization domain (NOD)‐like receptor pyrin domain containing 3 (NLRP3) inflammasome pathway's role in pancreatic ductal adenocarcinoma (PDAC) has been extensively studied, its implications in IPMN remain unexplored. This study aimed to investigate the prognostic significance of NLRP3 inflammasome‐related proteins across IPMN subtypes and their associations with tumor characteristics, with a secondary focus on comparing expression patterns in IPMN and PDAC. A cohort of 187 patients (100 IPMN and 87 PDAC) underwent high‐dimensional histopathological imaging using the multiplexed immunohistochemical consecutive staining on single slide method and a semi‐automated image analysis workflow. Expression levels of NLRP3, apoptosis‐associated speck‐like protein containing a caspase‐recruitment domain (ASC), caspase‐1, interleukin‐1 beta, interleukin‐18 (IL‐18), interleukin‐1 receptor antagonist, and interleukin‐18 binding protein (IL‐18BP) were evaluated and compared between IPMN and PDAC samples. The relationships between protein expression and tumor characteristics were examined. Principal component analysis distinguished between intestinal and nonintestinal clusters based on NLRP3‐associated proteins. Lower IL‐18 expression was linked to the intestinal subtype, while higher caspase‐1 was linked to the pancreatobiliary subtype. Elevated caspase‐1 and ASC expression were associated with invasiveness in IPMN. No significant correlation was found between the examined proteins and later‐stage tumor characteristics in invasive cases. The IL‐18/IL‐18BP ratio was an independent prognostic factor in invasive IPMN. Our findings highlight the prognostic significance of IL‐18 and the IL‐18/IL‐18BP ratio in invasive IPMNs. These results point to a complex regulation of NLRP3 inflammasome proteins, especially effector cytokines, in pancreatic neoplasms, which are strongly linked to subtype and prognosis.

## Introduction

According to the 2020 estimates of the Global Cancer Observatory, malignant pancreatic tumors rank 12th in global incidence and 7th in tumor‐related deaths, with a 5‐year survival rate of only 8% [1, 2]. Late tumor detection contributes to this poor prognosis, thus recognition and proper clinical interpretation of precancerous conditions of the pancreas is crucial for better survival [3]. Intraductal papillary mucinous neoplasm (IPMN) is the most common pancreatic cystic lesion known for malignant potential and early detectability by imaging techniques [4, 5, 6]. However, new molecular markers are needed to determine the risk of malignancy and select the fraction of patients that would benefit from surgery.

As an intracellular protein complex, the nucleotide‐binding oligomerization domain (NOD)‐like receptor pyrin domain containing 3 (NLRP3) inflammasome produces pro‐inflammatory cytokines in response to pathogens, homeostasis disruption, or cellular damage [7, 8]. Upon receiving a danger signal, NLRP3 undergoes conformational changes and forms NLRP3–apoptosis‐associated speck‐like protein containing a caspase‐recruitment domain (ASC)–caspase‐1 complex. This complex cleaves interleukin‐1 beta (IL‐1B) and interleukin‐18 (IL‐18) precursors into biologically active IL‐1B and IL‐18, which can drive inflammation [9].

Chronic pancreatitis is a well‐known risk factor for pancreatic cancer [10]. The role of the NLRP3 inflammasome in developing PDAC by creating chronic inflammation has already been investigated but the exact mechanisms still need to be clearly understood [11]. In the presence of the Kirsten rat sarcoma viral oncogene homolog (*KRAS*) mutation, chronic inflammation is likely to lead to pancreatic cancer through increased reactive oxygen species (ROS) production which causes deoxyribonucleic acid damage [12, 13].

Furthermore, the release of pro‐inflammatory cytokines derived by NLRP3 activation can activate inflammatory cancer‐associated fibroblasts, which event could contribute to the formation of desmoplastic stroma, enhance tumor cell survival, promote resistance to chemotherapy, and create an immunosuppressive microenvironment in pancreatic cancer [14, 15]. Additionally, the expression of NLRP3 inflammasome components is upregulated in cancer tissue and closely associated with the prognosis of surgically resectable pancreatic adenocarcinoma [16]. IPMN is also often associated with chronic pancreatitis and some studies have found an elevated IL‐1B level in cyst fluid compared to other pancreatic cystic neoplasms [17, 18, 19, 20]. Despite these findings, the exact role of inflammation, and more precisely the NLRP3 inflammasome, in tumor progression has not yet been investigated. Therefore, we aimed to explore the prognostic role and diverse interactions of NLRP3 inflammasome proteins and associated cytokines in pancreatic neoplasms.

## Materials and methods

### Ethical permission

All study protocols followed the ethical guidelines of the 1975 Declaration of Helsinki. Ethical approval was gained from the Hungarian Medical Research Council (approval number: BMEÜ/727‐1/2022/EKU).

### Samples and clinical data

The study cohort consisted of 187 patients (100 with IPMN and 87 with PDAC), who were over 18 years of age, underwent curative pancreas resection between 2008 and 2021, and had a pathology‐confirmed IPMN or PDAC. Patients with missing datasets, and other tumors were excluded. Clinical data including demographic information (age, gender), tumor location, type of resection, ductal involvement, subtype, histopathologic grade, TNM stage, perineural invasion, resection margin status, and overall survival were collected from the database of the Institute of Pathology, Forensic and Insurance Medicine, Semmelweis University, and Hungarian Cancer Registry. Representative tissue sections and their corresponding formalin‐fixed paraffin‐embedded (FFPE) tissue samples were collected from the institute's archive. Two pathologists reviewed the specimens to determine subtype, grade, areas of interest, and to exclude inappropriate samples. After the review, tissue microarrays (TMAs) were constructed. In invasive IPMN cases, four (two dysplastic and two invasive areas) were cored, while in noninvasive IPMN and PDAC cases two cores per case were spotted. Mucin profile and caudal‐type homeobox‐2 (CDX2) protein expression were determined based on the World Health Organization guidelines on the TMAs [21].

### Multiplex immunohistochemistry

Following the preparation of TMAs, 4‐μm sections were made of each TMA using a microtome and mounted onto glass slides. NLRP3, ASC, caspase‐1, IL‐1B, IL‐18, IL‐1RA, IL‐18BP, and pan‐cytokeratin (PanCK) proteins on one section, then mucin 1 (MUC1), mucin 2 (MUC2), mucin 5AC (MUC5AC), mucin 6 (MUC6), and CDX2 proteins on another section were sequentially stained using the multiplexed immunohistochemical consecutive staining on single slide technique [22]. Details of the staining protocol, including the reagents and antibodies used, and the parameters of the scanning can be found in Supplementary materials and methods and Tables S1–S4.

### Image analysis

After scanning, each slide was imported into QuPath 0.4.0, and color vector parameters for each stain were determined [23]. Then we annotated the individual core images and aligned those belonging to the same core with TrakEM2, ImageJ plugin [24]. The area of the tumor cells was segmented based on the PanCK stained images using Ilastik, then the preliminary segmentation masks were validated and corrected by two pathologists specializing in hepato‐pancreato‐biliary pathology [25]. The core images underwent deconvolution using staining‐specific color vectors. Then, for each core, we created the staining intensity histograms of the marker proteins in the previously segmented tumor region using ImageJ [26]. Based on the intensity histograms, we determined the Averaged Threshold Measure (ATM) score, as described by Choudhury *et al* [27]. For noninvasive IPMN and PDAC cases, the ATM score was calculated as the sum of intensity histograms from all associated cores. For invasive IPMN cases, ATM scores were calculated separately for cores containing dysplastic and invasive components. The ATM scores derived from the invasive component cores were used for subsequent analyses. The IL‐18/IL‐18BP ratio was calculated by dividing their standardized ATM score data. The exact workflow of image analysis and the mathematical formula of cytokine ratio determination are described in Supplementary materials and methods and Figure S1.

### Creation of pseudo‐fluorescent multiplex images

Color deconvolution was performed separately on images of individual proteins labeled with advanced 3‐amino‐9‐ethylcarbazole (AMEC) and hematoxylin, based on previously determined color vectors, using the Python HistomicsTK package. An arbitrary Look‐Up Table was assigned to the 8‐bit image of the AMEC channel, artificially recoloring the image. A composite image was then created from these recolored 8‐bit images using the Python scikit‐image library (supplementary material, Figure S2).

### Statistical analysis

Statistical analyses were performed using Python (Python Software Foundation, 2022, Beaverton, OR, USA) [28]. A *p* value less than 0.05 was considered statistically significant. For homogeneity testing, chi‐square test was used for categorical and Mann–Whitney *U* test was used for continuous variables. To compare protein expression based on different properties, Wilcoxon test was performed. Principal component analysis (PCA) was performed to select the factors that have the greatest impact on data and identify the relationships between them. Spearman correlation test was employed to assess the correlation among the expressions of proteins investigated. We used Cox proportional hazard model, Kaplan–Meier survival analysis, and log‐rank test to measure the prognostic value of each protein, cytokine ratio, and pathological factor. We used the overall survival time, calculated from the day of diagnosis and expressed in months.

## Results

### Characteristics of the cohort

The mean age of patients was 66.7 years (males: 66 years, females: 67 years) (Table 1). Of the IPMN cases, 32 showed invasive histopathology, while 68 were dysplastic without invasion. Invasive cases were significantly more often the main duct type, pancreatobiliary subtype, and MUC1 positive. Considering all invasive cases, significantly higher stage, grade, lymph node positivity, and perineural invasion frequency were observed in PDAC cases compared to invasive IPMN cases.

**Table 1:** Clinical characteristics of IPMN and PDAC cases

	Noninvasive IPMN, *N* = 68	Invasive IPMN, *N* = 32	*p*		Invasive IPMN, *N* = 32	PDAC, *N* = 87	*p*
**Age** **(years)** *	68 [38, 83]	71 [41, 80]	0.18	**Age (years)** *	71 [41, 80]	68 [37, 86]	0.43
**Sex**				**Sex**			
Male	29 (42.6%)	16 (50%)	0.64	Male	16 (50%)	33 (37.9%)	0.33
Female	39 (57.4%)	16 (50%)		Female	16 (50%)	54 (62.1%)	
**Location**				**Location**			
Head	47 (69.1%)	18 (56.2%)	0.23	Head	18 (56.2%)	62 (71.3%)	0.44
Body	10 (14.7%)	3 (9.4%)		Body	3 (9.4%)	5 (5.8%)	
Tail	6 (8.8%)	6 (18.8%)		Tail	6 (18.8%)	9 (10.3%)	
Diffuse	5 (7.4%)	5 (15.6%)		Diffuse	5 (15.6%)	11 (12.6%)	
**Type of surgery**				**Type of surgery**			
Pancreatoduodenectomy	35 (51.5%)	15 (46.9%)	0.29	Pancreatoduodenectomy	15 (46.9%)	48 (55.2%)	0.07
Distal pancreatectomy	15 (22.1%)	3 (9.4%)		Distal pancreatectomy	3 (9.4%)	16 (18.4%)	
Total pancreatectomy	16 (23.5%)	13 (40.6%)		Total pancreatectomy	13 (40.6%)	15 (17.2%)	
Other	2 (2.9%)	1 (3.1%)		Other	1 (3.1%)	8 (9.2%)	
**Overall survival (months)** *	48.5 [1, 164]	24 [0, 128]	**0.0004**	**Overall survival (months)** *	24 [0, 128]	17 [0, 88]	0.14
**Duct type**				**Resection margin**			
Main	6 (8.8%)	11 (34.4%)	**0.01**	R0	19 (59.4%)	52 (59.7%)	0.83
Mixed	42 (61.8%)	16 (50.0%)		R1	13 (40.6%	34 (39.1%)	
Branch	20 (29.4%)	5 (15.6%)		RX	0	1 (1.2%)	
**Subtype**				**T**			
Pancreatobiliary	5 (7.4%)	19 (59.4%)	**<0.0001**	T1	9 (28.1%)	2 (2.3%)	**0.0002**
Gastric	34 (50%)	6 (18.8%)		T2	9 (28.1%)	24 (27.6%)	
Intestinal	29 (42.6%)	7 (21.8%)		T3	14 (43.8%)	59 (67.8%)	
**Dysplasia**				T4	0	2 (2.3%)	
Low	48 (70.6%)	–	**–**	**Nodal status**			
High	20 (29.4%)			Negative	13 (40.6%)	16 (18.4%)	**0.03**
**MUC1 expression**				Positive	19 (59.4%)	69 (79.3)	
Positive	31 (45.6%)	31 (96.9%)	**<0.0001**	NA	0	2 (2.3%)	
Negative	37 (54.4%)	1 (3.1%)		**M**			
**MUC2 expression**				M0	30 (93.8%)	84 (96.6%)	0.87
Positive	29 (42.6%)	8 (25%)	0.14	M1	2 (6.2%)	3 (3.4%)	
Negative	39 (57.4%)	24 (75%)		**Modified AJCC stage (8th)**			
**MUC5 expression**				IA	7 (21.9%)	0	**0.0006**
Positive	60 (88.2%)	26 (81.3%)	0.53	IB	2 (6.2%)	5 (5.7%)	
Negative	8 (11.8%)	6 (18.7%)		IIA	12 (37.5%)	27 (31%)	
**MUC6 expression**				IIB	5 (15.6%)	28 (32.2%)	
Positive	42 (61.8%)	16 (50%)	0.53	III	4 (12.5%)	23 (26.4%)	
Negative	26 (38.2%)	16 (50%)		IV	2 (6.3%)	2 (2.3%)	
**CDX2 expression**				NA	0	2 (2.3%)	
Positive	44 (64.7%)	18 (56.3%)	0.55	**Grade**			
Negative	24 (38.2%)	14 (43.7%)		G1	14 (43.8%)	4 (4.6%)	**<0.0001**
				G2	14 (43.8%)	49 (56.3%)	
				G3	4 (12.4%)	34 (39.1%)	
				**Perineural invasion**			
				Yes	19 (59.4%)	77 (88.5%)	**0.001**
				No	7 (21.9%)	6 (6.9%)	
				NA	6 (18.7%)	4 (4.6%)	
				**Phenotype**			
				Tubular	25 (78.1%)	–	**–**
				Intestinal	7 (21.9%)		

The table compares noninvasive and invasive IPMN cases (left panel) and invasive IPMN versus PDAC cases (right panel). Tumor staging parameters (T‐stage, grade) follow the TNM Classification of Malignant Tumors 8th edition. The ‘Other’ category in surgical procedures encompasses enucleation and subtotal pancreatectomy. Statistical significance was determined using chi‐square test for categorical variables and Mann–Whitney *U* test for continuous variables, with *p* < 0.05 considered significant. Significant *p* values are shown in bold font.

NA, not available.

* Age is presented as median (range). All other variables are shown as absolute numbers with percentages in parentheses.

### Impact of protein expression patterns on prognostic factors of IPMN

**Figure 1:**
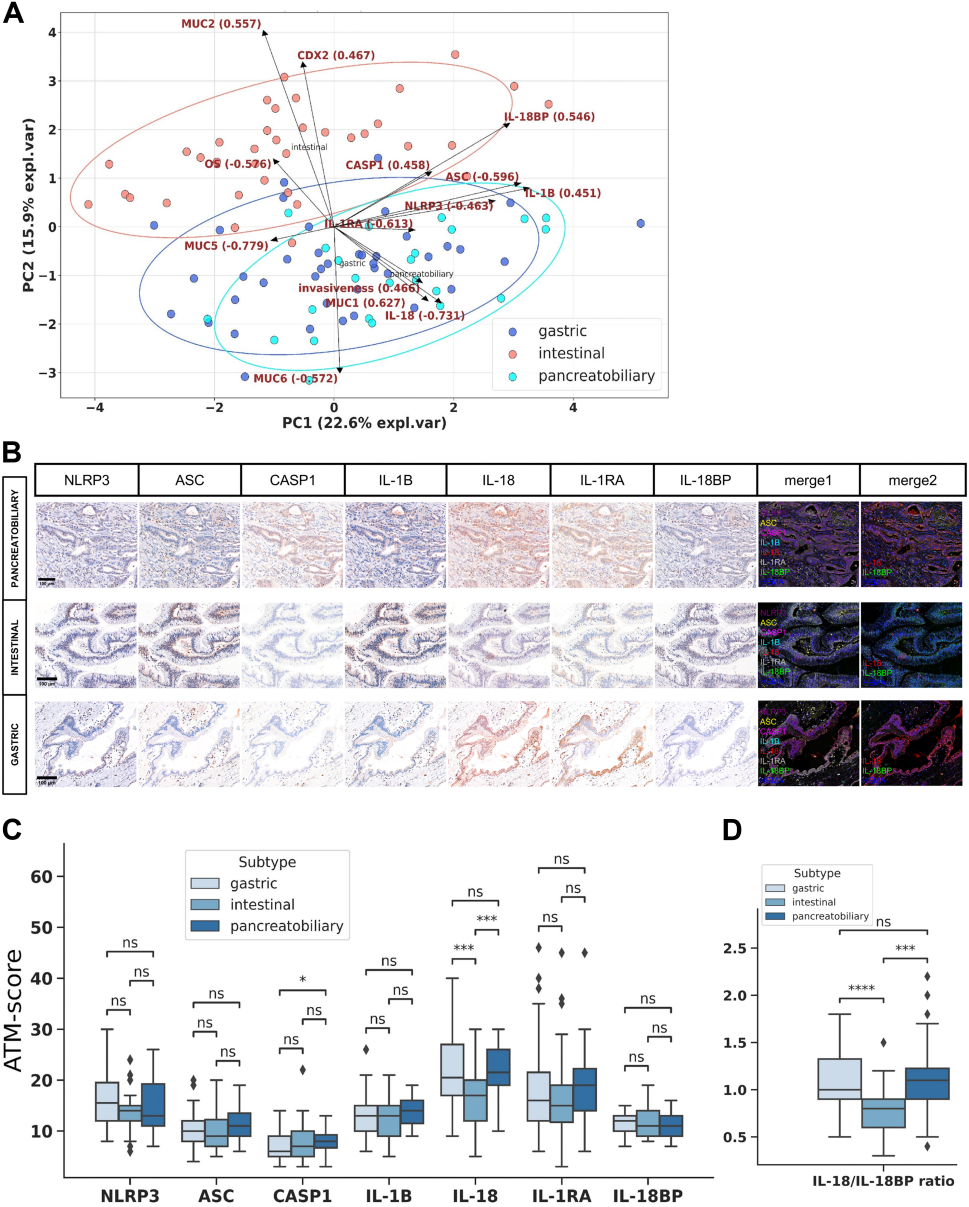
Associations between IPMN subtypes and the expression of NLRP3 inflammasome proteins. (A) PCA biplot illustrating the distribution of IPMN subtypes based on overall survival, invasiveness, and the expression of mucins and inflammasome proteins. Loading vectors represent the contribution of each variable to the principal components (PCs). The first two PCs explain 38.5% of the total variance. Data points represent individual IPMN cases, with colors denoting subtypes. Ellipses indicate 95% CIs for a bivariate *t*‐distribution fitted to data points of each subtype. (B) Immunohistochemical staining patterns based on representative images, showing AMEC and hematoxylin counterstaining, merged fluorescence‐like images of all examined proteins (merge1), and a focused merge showing IL‐18, IL‐18BP, and hematoxylin counterstaining (merge2) (scale bar: 100 μm). (C) Quantitative comparison using ATM scores between the subtypes (*N*_gastric_ = 40, *N*_intestinal_ = 26, *N*_pancreatobiliary_ = 24). (D) Quantitative comparison of IL‐18/IL‐18BP ratios based on IPMN subtypes. In panels (C) and (D), lines represent medians, boxes represent interquartile ranges, and whiskers extend 1.5 times the interquartile ranges. Points represent outliers. Statistical analysis: Wilcoxon test, markings: ns, *p* > 0.05; *p < 0.05; **p < 0.01; ***p < 0.001; ****p < 0.0001. CASP1, caspase‐1; HEMA, hematoxylin; OS, overall survival.

**Figure 2:**
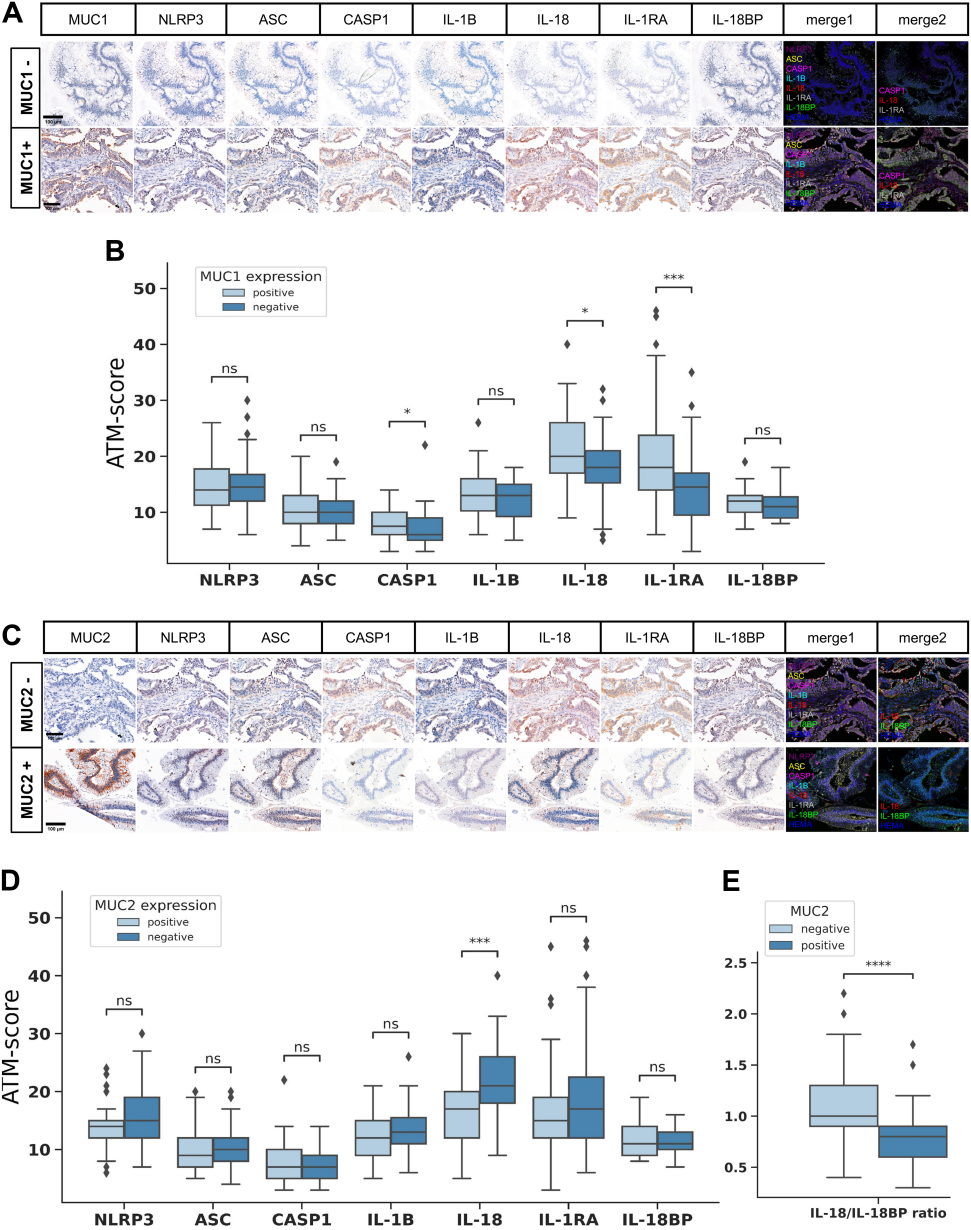
Associations between MUC1 and MUC2 profiles and the expression of NLRP3 inflammasome proteins in IPMN. (A) Immunohistochemical staining patterns of MUC1‐positive and ‐negative cases based on representative images, showing AMEC and hematoxylin counterstaining, merged fluorescence‐like images of all examined proteins (merge1), and a focused merge showing CASP1, IL‐18, IL‐1RA, and hematoxylin counterstaining (merge2) (scale bar: 100 μm). (B) Quantitative comparison of ATM scores based on MUC1 expression (*N*_positive_ = 62, *N*_negative_ = 38). (C) Immunohistochemical staining patterns of MUC2‐positive and ‐negative cases based on representative images, showing AMEC and hematoxylin counterstaining, merged fluorescence‐like images of all examined proteins (merge1), and a focused merge showing IL‐18, IL‐18BP, and hematoxylin counterstaining (merge2) (scale bar: 100 μm). (D) Quantitative comparison of ATM scores based on MUC2 expression (*N*_positive_ = 37, *N*_negative_ = 63). (E) Quantitative comparison of IL‐18/IL‐18BP ratios based on MUC2 expression in IPMN cases. In panels (B), (D), and (E), lines represent medians, boxes represent interquartile ranges, and whiskers extend 1.5 times the interquartile ranges. Points represent outliers. Statistical analysis: Wilcoxon test, markings: ns, *p* > 0.05; *p < 0.05; **p < 0.01; ***p < 0.001; ****p < 0.0001. CASP1, caspase‐1; HEMA, hematoxylin.

**Figure 3:**
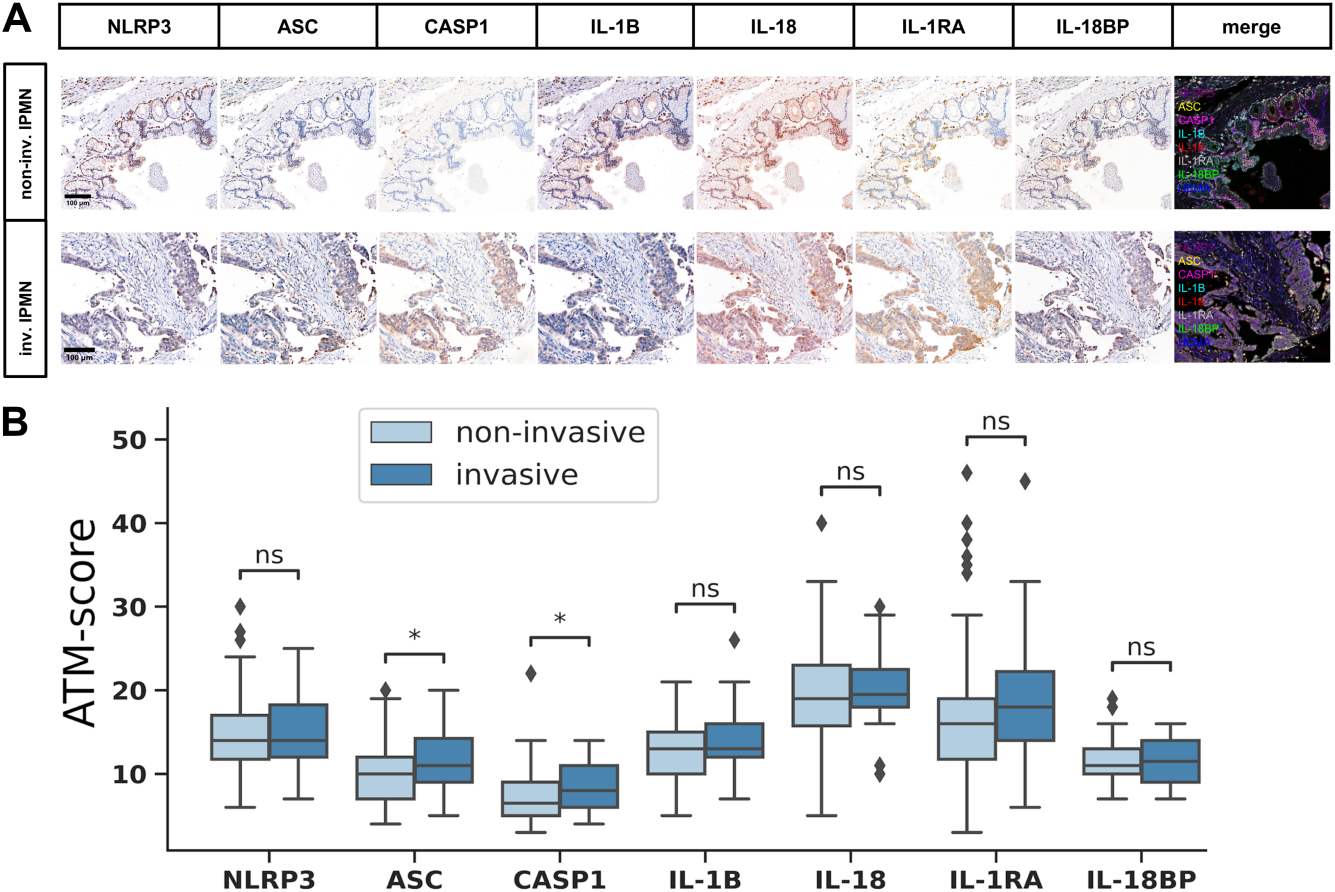
NLRP3‐associated protein expression patterns in invasive and noninvasive IPMN cases. (A) Immunohistochemical staining patterns of noninvasive and invasive IPMN cases based on representative images, showing AMEC and hematoxylin counterstaining and merged fluorescence‐like images of all examined proteins (merge) (scale bar: 100 μm). (B) Quantitative comparison of ATM scores in noninvasive (*N* = 68) and invasive IPMN (*N* = 32) cases. Lines represent the medians, boxes represent interquartile ranges, and whiskers extend 1.5 times the interquartile ranges. Points represent outliers. Statistical analysis: Wilcoxon test, markings: ns, *p* > 0.05; *p < 0.05; **p < 0.01; ***p < 0.001; ****p < 0.0001. non‐inv., noninvasive; inv., invasive; CASP1, caspase‐1; HEMA, hematoxylin.

We investigated how the known pathological phenotypic factors of IPMN, such as ductal involvement, subtype, expression of mucins (MUC1, MUC2, MUC5AC, MUC6) and CDX2, and invasiveness, are associated with the expression pattern of the NLRP3 inflammasome proteins. No significant differences were found in protein expression levels between IPMN cases with main duct, branch duct, or mixed ductal involvement (supplementary material, Figure S3A). We performed exploratory factor analysis to investigate the relationship between invasiveness, the subtypes of IPMN, expression of mucins, CDX2, and NLRP3 inflammasome proteins (Figure 1A). The loading vectors of IL‐18 expression, MUC1 positivity, and invasiveness were closely related, while the vectors of the other tested proteins were not significantly separated. Based on the biplot, the intestinal and nonintestinal samples were separated. Consistent with this, we observed a significantly lower level of IL‐18 and a slightly higher level of IL‐18BP expression in the intestinal subtype of IPMN compared to the nonintestinal subtypes. Additionally, cases of the pancreatobiliary subtype showed higher expression of caspase‐1 compared to those of the gastric subtype (Figure 1B,C). As IL‐18 and IL‐18BP showed contrasting expression patterns, we explored the correlation between the IL‐18/IL‐18BP ratio and the samples' subtypes and mucin expression profiles. The IL‐18/IL‐18BP ratio was significantly decreased in intestinal type IPMNs and demonstrated a more pronounced separation between the subtypes than IL‐18 expression alone (Figure 1D). We then investigated the more detailed relationship between the protein markers that define the IPMN phenotype and the expression of each inflammasome protein and IL‐18/IL‐18BP ratio. MUC1‐positive cases had significantly higher caspase‐1, IL‐18, and IL‐1RA expression (Figure 2A,B), while MUC2‐positive cases had significantly lower IL‐18 expression and IL‐18/IL‐18BP ratio (Figure 2C–E). There were no significant differences in the examined protein expressions based on MUC5AC, MUC6, and CDX2 positivity (supplementary material, Figure S3B–D). Examining the association of NLRP3 inflammasome proteins with invasiveness, significantly higher caspase‐1 and ASC expression was observed in invasive than noninvasive IPMN cases (Figure 3). Analyzing protein expression relative to dysplasia, we observed significant differences in ASC and caspase‐1 expression between invasive and noninvasive cases which could be attributed to disparities between low‐grade and invasive samples (supplementary material, Figure S4A). No distinctions were identified in protein expression between low‐ and high‐grade IPMN cases for any examined proteins. When specifically comparing dysplastic versus invasive components of the invasive IPMN cases, no statistically significant differences were observed in the expression levels of any of the analyzed proteins (supplementary material, Figure S4B,C). Then we compared invasive and noninvasive cases separately within intestinal and nonintestinal subtypes relying on the PCA separation. In intestinal cases, NLRP3, ASC, and IL‐1B showed slight increase, while IL‐1RA was significantly elevated in invasive cases (supplementary material, Figure S5A,B). Conversely, in nonintestinal cases, higher caspase‐1 expression was observed in invasive cases (supplementary material, Figure S5C,D).

### Effect of the analyzed proteins on the biological behavior of invasive cases

**Figure 4:**
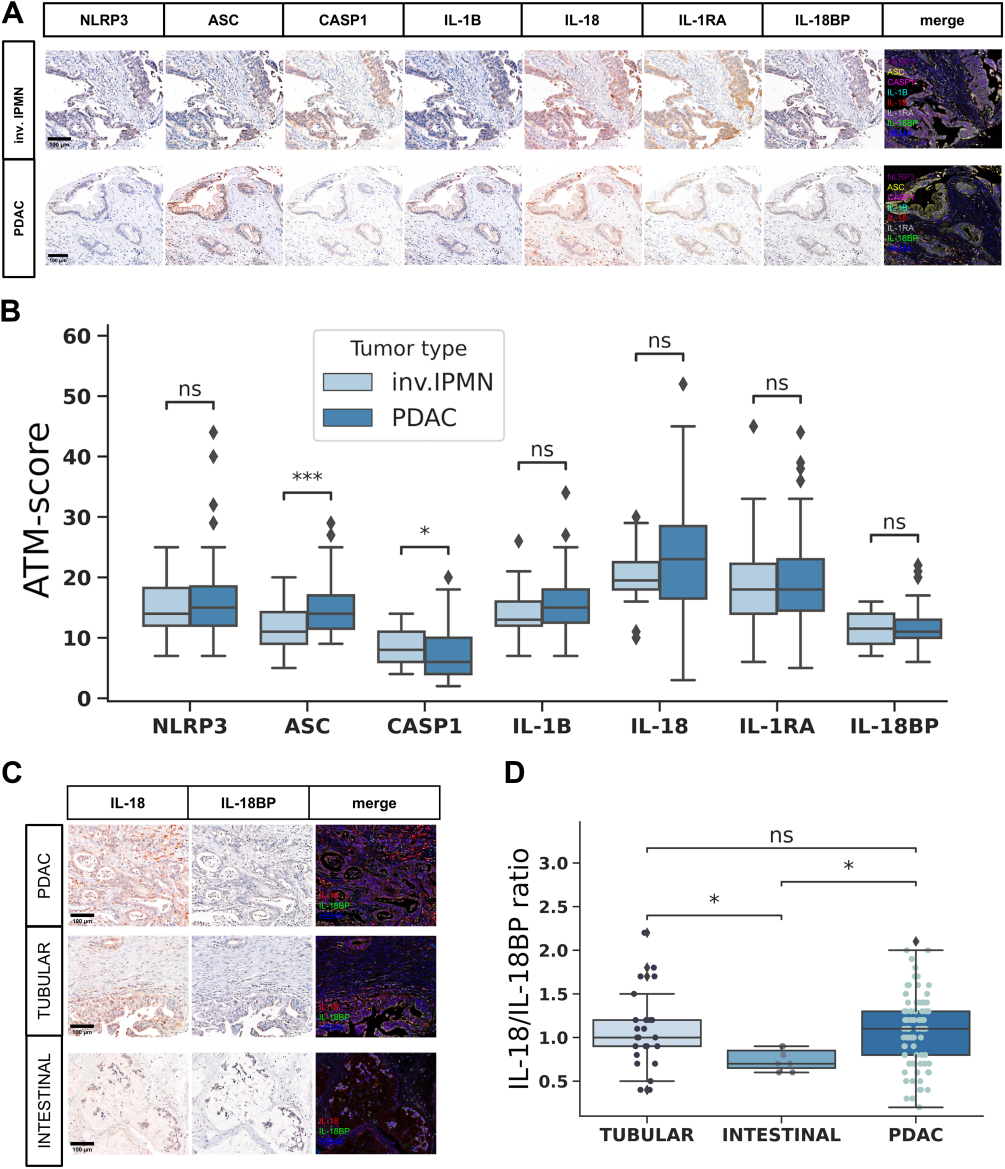
NLRP3‐associated protein expression patterns in invasive IPMN and PDAC cases. (A) Immunohistochemical staining patterns of invasive IPMN and PDAC cases based on representative images, showing AMEC and hematoxylin counterstaining and merged fluorescence‐like images of all examined proteins (merge) (scale bar: 100 μm). (B) Quantitative comparison of ATM scores in invasive IPMN (*N* = 32) and PDAC (*N* = 87) cases. (C) Immunohistochemical staining patterns of PDAC tubular and intestinal carcinoma types based on representative images, showing AMEC and hematoxylin counterstaining and merged fluorescence‐like images of IL‐18, IL‐18BP, and hematoxylin counterstaining (merge) (scale bar: 100 μm). (D) Quantitative comparison of IL‐18/IL‐18BP ratios in PDAC (*N* = 87), tubular (*N* = 25), and intestinal‐type (*N* = 7) carcinoma cases. In panels (B) and (D), lines represent medians, boxes represent interquartile ranges, and whiskers extend 1.5 times the interquartile ranges. Points represent individual data points in panel (D). Statistical analysis: Wilcoxon test, markings: ns, *p* > 0.05; *p < 0.05; **p < 0.01; ***p < 0.001; ****p < 0.0001. CASP1, caspase‐1; HEMA, hematoxylin; inv., invasive.

**Figure 5:**
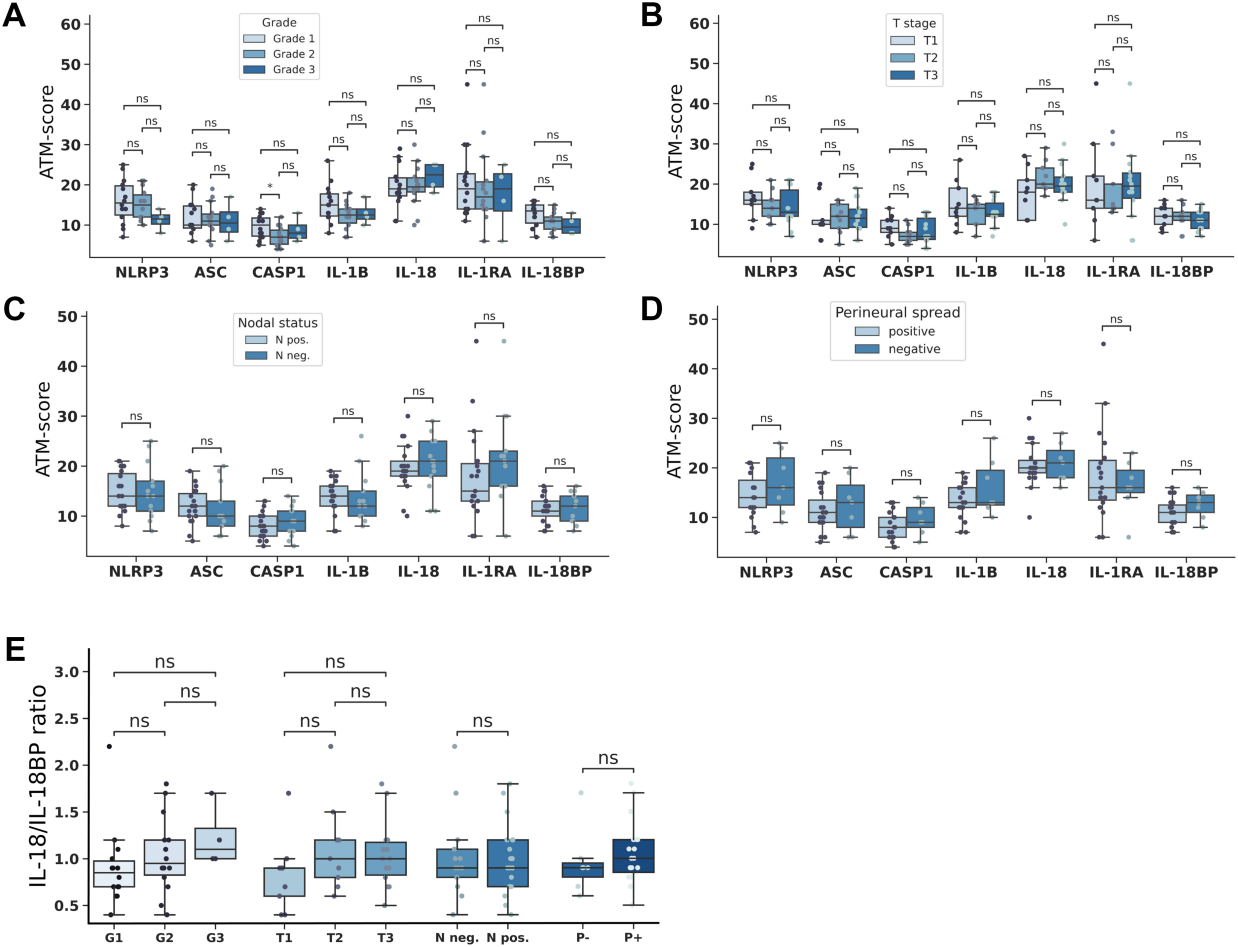
Relationship between NLRP3 inflammasome proteins and pathological characteristics of invasive IPMN. (A–D) Association between pathological prognostic factors, including (A) grade (*N*_grade1_ = 14, *N*_grade2_ = 14, *N*_grade3_ = 4), (B) T stage (*N*_T1_ = 9, *N*_T2_ = 9, *N*_T3_ = 14), (C) nodal status (*N*_Npos_ = 19, *N*_Nneg_ = 13), and (D) perineural invasion (*N*_positive_ = 19, *N*_negative_ = 7), with ATM scores of the examined proteins. (E) Association between pathological prognostic factors and IL‐18/IL‐18BP ratio. Lines represent medians, boxes represent interquartile ranges, and whiskers extend 1.5 times the interquartile ranges. Points represent individual data points. Statistical analysis: Wilcoxon test, markings: ns, *p* > 0.05; *p < 0.05; **p < 0.01; ***p < 0.001; ****p < 0.0001. CASP1, caspase‐1; neg., negative; P−, perineural invasion negative; P+, perineural invasion positive; pos., positive.

Comparing invasive IPMN and PDAC based on the individual protein expressions, significantly lower ASC and higher caspase‐1 expression was observed in invasive IPMN cases (Figure 4A,B). We have previously described that the IL‐18/IL‐18BP ratio differs significantly between intestinal and nonintestinal cases (Figure 1D). Therefore, we compared this cytokine ratio in PDAC cases and, separately, in the two invasive phenotypes (tubular and intestinal) of IPMN cases. The IL‐18/IL‐18BP ratio was significantly higher in the tubular carcinoma and PDAC cases compared to the intestinal‐type carcinomas (Figure 4C,D). Then we performed the Spearman correlation test to examine the relationship between the inflammasome proteins and the characteristics that determine the progression and biological behavior of invasive tumors, such as lymph node status, T stage, grade, and perineural spread (supplementary material, Figure S6A). Our findings revealed a lack of significant correlation between the expression of the tested proteins and the aforementioned pathological prognostic factors considering all the invasive cases. Then we examined invasive IPMN and PDAC cases separately. Significantly higher caspase‐1 expression was observed in grade 1 compared to grade 2 IPMN cases (Figure 5A). In PDAC cases, however, grade did not correlate with the expression of any NLRP3‐associated proteins tested (supplementary material, Figure S6B). In both PDAC and invasive IPMN cases, there were no significant differences in the expression of the tested proteins depending on T stage, lymph node positivity, or perineural invasion (Figure 5B–D and supplementary material, Figure S6C–E). Neither PDAC nor invasive IPMN cases showed significant differences in the IL‐18/IL‐18BP ratio in terms of any prognostic factor examined (Figure 5E and supplementary material, Figure S6F).

### Impact of protein expression patterns on overall survival

**Figure 6:**
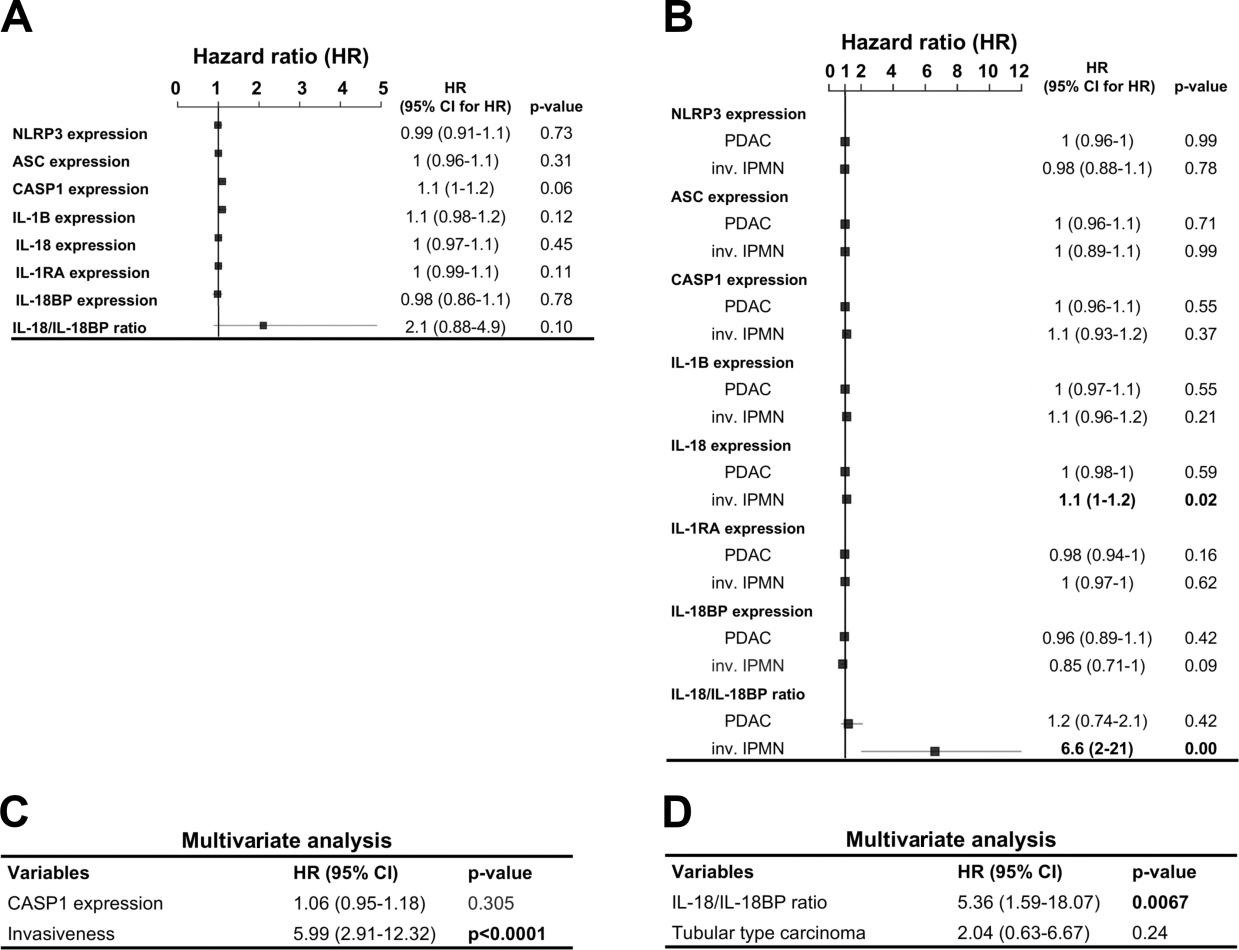
Impact of the NLRP3 inflammasome proteins on overall survival in pancreatic neoplasms. (A and B) Cox proportional hazards models based on ATM scores measuring the impact of inflammasome proteins on overall survival in (A) IPMN and (B) invasive cases, including IPMN and PDAC separately. (C) Multivariate Cox proportional hazards model investigating the independent prognostic role of caspase‐1 in IPMN cases. (D) Multivariate Cox proportional hazards model investigating the IL‐18/IL‐18BP ratio in invasive IPMN cases. When *p* < 0.05, differences were considered significant. CASP1, caspase‐1; CI, confidence interval; HR, hazard ratio; inv., invasive.

We used Cox proportional hazard modeling and Kaplan–Meier analysis to evaluate the impact of pathological prognostic factors and specific proteins, including NLRP3, ASC, caspase‐1, IL‐1B, IL‐18, IL‐1RA, IL‐18BP, as well as the IL‐18/IL‐18BP ratios, on survival of patients with IPMN and PDAC. In IPMN cases, main duct type, MUC1 positivity, pancreatobiliary subtype, and invasiveness were significantly associated with an increased hazard, while the branch duct type reduced the hazard ratio (supplementary material, Figure S7A). Among the evaluated proteins and cytokine ratios, caspase‐1 expression tended to show an association with the increased hazard of death (Figure 6A). When comparing hazard factors regarding grade, T‐stage, nodal status, distant metastasis, positive resection margin, and perineural invasion in PDAC and invasive IPMN cases, only high‐grade showed a significant increase in hazard in PDAC (supplementary material, Figure S7B). According to Kaplan–Meier analysis, tubular carcinomas in invasive IPMN cases showed a trend toward worse survival than intestinal type carcinomas (supplementary material, Figure S7C). We then examined the impact of proteins and cytokine ratios on survival in PDAC and invasive IPMN separately (Figure 6B). Higher IL‐18 expression, and particularly a high IL‐18/IL‐18BP ratio were associated with a significantly increased hazard in invasive IPMN cases. For PDAC, none of the investigated proteins or cytokine ratios showed a significant association with the hazard ratio. Multivariate Cox analysis revealed that caspase‐1 was not an independent prognostic factor in IPMN cases (Figure 6C). However, the IL‐18/IL‐18BP expression ratio emerged as an independent prognostic factor in invasive IPMN cases (Figure 6D).

## Discussion

The survival rate for pancreatic cancer remains low at 8% after 5 years [2]. It is crucial to detect the tumor early or identify and interpret precancerous lesions correctly. IPMN is the most frequent precancerous lesion of the pancreas, but determining its transformation into malignancy is difficult [29]. Chronic, NLRP3 inflammasome‐driven inflammation is potentially significant in developing pancreatic cancer [12, 30]. However, the role of its major effector cytokines (IL‐1B and IL‐18) and their known antagonists (IL‐1RA and IL‐18BP) in carcinogenesis or malignant transformation of IPMN remains unclear. Therefore, this study investigated the protein expression of NLRP3, ASC, caspase‐1, IL‐1B, IL‐18, IL‐1RA, and IL‐18BP on tumor cells by utilizing multiplexed immunohistochemistry on PDAC and IPMN samples, examining their association with pathological features and prognoses. Regarding our cohort, relative to pre‐malignant cases, the higher prevalence of main‐duct type, MUC1‐positive, pancreatobiliary subtype tumors in invasive IPMN cases, aligns with existing literature [31]. Additionally, our data supports that invasive IPMNs discovered at an earlier stage have better differentiation and more indolent histopathological features compared to conventional PDAC cases, which is also consistent with established scientific observations [32, 33]. These results affirm the representativeness of our cohort.

Our multiparametric analysis revealed a distinct clustering of intestinal and nonintestinal subtypes of IPMN, primarily influenced by differences in MUC1, MUC2, IL‐18, and IL‐18BP protein expression. MUC2‐positive, intestinal subtype IPMNs showed significantly lower IL‐18 expression and slightly higher IL‐18BP levels, resulting in a marked difference in the IL‐18/IL‐18BP ratio compared to nonintestinal subtypes. However, there were no significant differences in the expression of these cytokines between the pancreatobiliary and gastric subtypes. These observations are consistent with the results of Liffers *et al* who described that intestinal IPMNs have a distinct genetic landscape and different epigenetic profile compared to pancreatic intraepithelial neoplasia and gastric IPMNs [34]. This genetic and epigenetic difference also seems to be manifested in the expression of IL‐18 and IL‐18BP cytokines. IL‐18 expression requires the co‐activation of nuclear factor kappa‐light‐chain enhancer of activated B cells (NF‐κB) and the type I interferon pathway [35]. An explanation might be the higher guanine nucleotide‐binding protein, alpha‐stimulating activity polypeptide (GNAS) and lower *KRAS* mutation frequency in intestinal subtype IPMNs [36, 37]. *GNAS* mutation, through protein kinase A activation, inhibits NF‐κB and might lead to reduced IL‐18 expression, while *KRAS* mutation can increase IL‐18 expression by activating NF‐κB [38, 39, 40, 41]. Also, Reggiardo *et al* demonstrated that *KRAS* mutation induces interferon‐stimulated gene signature in lung cancers [42]. This suggests that mutations associated with different subtypes and a difference in type I interferon stimulus may reverse the regulation of IL‐18 and IL‐18BP expression.

In our analysis of NLRP3‐associated protein expression patterns in relation to dysplasia, we observed that ASC and caspase‐1 expression levels were significantly elevated in invasive cases. Notably, the most pronounced differences were evident when comparing low‐grade versus invasive cases. However, no significant differences were detected between high‐grade and invasive cases, nor between the dysplastic and invasive components of invasive IPMN cases. These findings suggest that the increased expression of these proteins is associated with earlier mutational events and potentially reaches a plateau at the high‐grade dysplasia or carcinoma *in situ* stage. This observation aligns with the concept of stepwise progression in IPMN and with studies on other precancerous lesions, where inflammatory processes are often initiated early in neoplastic transformation, with molecular alterations accumulating gradually during the transition from low‐grade dysplasia to invasive carcinoma [43]. When we examined the association of inflammasome proteins with dysplasia in intestinal and nonintestinal cases separately, our results suggested potential differences in both the tumor development pathways and involvement of NLRP3‐associated proteins in malignant transformation. Observing a trend toward elevated NLRP3, ASC, IL‐1B, and significantly higher IL‐1RA expression in invasive intestinal compared to noninvasive cases suggests that higher NLRP3 inflammasome priming is associated with the development of malignancy in intestinal IPMN cases. It is well‐documented in the literature that intestinal metaplasia can develop and lead to adenocarcinoma in various chronic inflammatory conditions, such as Barrett's esophagus and *Helicobacter pylori*‐associated gastritis [44, 45, 46]. In these conditions, the activation of the NF‐κB signaling pathway plays an important role in the development of chronic inflammation, leading to increased expression of the NLRP3 inflammasome [47, 48]. Furthermore, these lesions were observed more frequently in patients with intestinal IPMN [44, 45, 46]. The literature data and our results further support the observation that chronic inflammation, through activation of the NF‐κB signaling pathway, may play a role in the malignant transformation of intestinal‐type IPMNs. However, this seems to contradict the hypothesized effect of GNAS in reducing NF‐κB activation. The reason may be that the *GNAS* mutation occurs relatively early, while NF‐κB activation contributes to later events. Several research groups have investigated the level of IL‐1B protein in the cyst fluid of IPMNs as a potential marker of malignancy; however, the results are controversial [17, 18, 19]. We should mention that none of the studies provided information on the subtype composition of IPMN cases. Based on our findings, the differing number of intestinal‐type IPMNs in the various study cohorts could be a factor contributing to the differing outcomes. We also must admit that the low number of invasive intestinal cases in our cohort limits this assumption.

In the nonintestinal cluster, significantly higher caspase‐1 expression was observed in the cases with MUC1 positivity, invasive phenotype, and pancreatobiliary subtype compared to the noninvasive, gastric subtype cases. Also, no significant difference was observed in IL‐18 and IL‐18BP expression. Higher expression of caspase‐1 has been described in PDAC cases compared to normal pancreatic tissue; however, it is unclear whether caspase‐1 expression is a cause or a consequence of malignant progression [49]. One explanation may be the higher MUC1 expression observed in pancreatobiliary and invasive cases, which can activate the interferon‐gamma signaling pathway in tumor cells, potentially leading to increased caspase‐1 expression [50, 51]. This hypothesis is reinforced by the higher levels of caspase‐1, IL‐18, and IL‐1RA observed in MUC1‐positive IPMN cases, which is consistent with the protein expression pattern observed in MUC1‐expressing PDAC, as demonstrated by several studies, including our findings [49, 52, 53]. Although our study's limitations do not allow precise determination of active caspase‐1 levels, Yang *et al* noted that 60% of PDAC samples showed caspase‐1 activity [49]. In this context, we can hypothesize that active caspase‐1 levels are also elevated in invasive IPMN cases, which may directly lead to increased levels of biologically active IL‐18. IL‐18 can create an immunosuppressive microenvironment, as seen in PDAC, contributing to malignant transformation [54]. Our results support the hypothesis that some gastric IPMNs can progress to tubular adenocarcinoma through pancreatobiliary morphology with IL‐18‐driven immunosuppression as a possible mechanism for this progression [55].

When comparing the expression of NLRP3‐associated proteins in the invasive cases, high ASC and lower caspase‐1 expression were observed in PDAC compared to invasive IPMN. In contrast, no significant difference was observed in the expression of other proteins. Several studies have demonstrated higher ASC and caspase‐1 expression in PDAC than in normal pancreas tissue. Additionally, higher expression of these proteins is associated with lower grade and tumor stage in PDAC [16, 56]. This aligns with our finding that grade 1 invasive IPMN samples show higher caspase‐1 expression than grade 2 cases. These results suggest that the higher caspase‐1 expression in invasive IPMN is due to the lower grade and stage of these cases. However, these observations seem contradictory to ASC expression. Koizumi *et al* discovered that the overexpression of ASC in PDAC can induce cell cycle upregulation by activating cyclin D1, resulting in enhanced cancer cell survival [56]. Our findings propose that this process is less active in invasive IPMN cases, potentially explaining the more indolent behavior compared to PDAC [32]. Higher IL‐18/IL‐18‐BP ratios were observed in tubular adenocarcinomas and PDACs compared to intestinal type adenocarcinomas, while no significant difference was observed between the other two groups. Mino‐Kenudson *et al* also observed similarities between tubular carcinoma and PDAC, while both are significantly different from intestinal‐type carcinoma. Along with our results, it suggests a link between differences in interleukin regulation and the level of malignancy in IPMN [57].

No significant correlation was observable between NLRP3‐associated protein expressions and tumor stage, nodal status, and perineural invasion in invasive cases. These findings suggest that the pathways causing inflammasome protein expression are not linked to tumor cell appearance and behavior in later‐stage tumors. This conflicts with the findings of Zheng and Liu, who discovered higher expression of NLRP3, ASC, caspase‐1, and IL‐1B in lymph node‐positive PDAC cases; however, it is worth noting that their study included only stage I–II tumors and segmentation of tumor cells from stroma was not performed [16].

Furthermore, the impact of important pathological factors, proteins (NLRP3, ASC, caspase‐1, IL‐1B, IL‐18, IL‐1RA, IL‐18BP) and IL‐18/IL‐18BP cytokine ratio on survival in IPMN and PDAC was investigated. The negative prognostic role of main duct involvement, MUC1 positivity, pancreatobiliary subtype, invasiveness, and the positive role of branch duct involvement in IPMN is consistent with the literature [46, 58, 59, 60]. NLRP3, ASC and IL‐1B hold no prognostic value either in IPMN or PDAC, which conflicts with the results of Zheng and Liu, who described a negative prognostic role for these proteins in PDAC [16]. This is probably because their study focused on stage I–II tumors exclusively. Elevated caspase‐1 expression was ruled out as an independent prognostic factor. This may be due to its strong correlation with pancreatobiliary subtype, MUC1 positivity, and invasiveness, based on our results. IL‐18 and in a more significant manner IL‐18/IL‐18BP protein expression ratio seemed to be an independent prognostic factor in invasive IPMNs. In PDAC, high IL‐18 expression was reported as a prognostic factor for poor outcome [52]. However, the mechanism behind this appears to be more convoluted. According to the report by Guo *et al*, NF‐κB activation is essential for cancer progression in PDAC. If the NF‐κB pathway is active, IL‐18 may promote tumor progression, while blocking NF‐κB allows IL‐18 to exert an antitumor effect [61]. Furthermore, according to Lutz *et al*, elevated IL‐18 receptor signaling significantly reduces T‐cell mediated cytotoxicity, as tumor‐derived IL‐18 can lead to cytotoxic T‐cell exhaustion [62]. Additionally, by promoting PD‐L1 signaling, IL‐18 suppresses NK‐cell surveillance, further deteriorating antitumor cellular immune response [63, 64]. Similar mechanisms are plausible in invasive IPMN. The observed beneficial effect of elevated IL‐18BP relative to IL‐18 (lower IL‐18/IL‐18BP ratio) suggests a significant counteraction to the deteriorating effect of IL‐18. However, further investigation is necessary to reveal the exact mechanisms, as no literature data currently exists on this topic.

This study was conducted on FFPE samples of IPMN and PDAC, thus experiments were carried out with commercially available and tested primary antibodies. It is not possible to make a clear statement whether interleukins and caspase‐1 are activated as their available antibodies can show reactivity with both pre and active isoforms.

In conclusion, the expression of IL‐18 and IL‐18BP proteins clearly distinguishes between intestinal and nonintestinal subtypes in IPMN. Our results also suggest different mechanisms of malignant transformation in these two clusters. In intestinal IPMN, enhanced NLRP3 priming appears to play a role in malignant transformation whereas, in nonintestinal cases, caspase‐1 activation and IL‐18‐driven immunosuppression may contribute to malignant degeneration. We confirmed that NLRP3 inflammasome proteins do not have a significant role in late tumor progression. IL‐18/IL‐18BP ratio was found to be a significant negative independent prognostic factor in invasive IPMN. This highlights the importance of understanding cytokine regulation in prognosis and potential treatment of IPMN and pancreatic cancer. Further investigation is necessary to elucidate these mechanisms and their implications for therapy completely.

## Author contributions statement

KN and AB conceived the study. KB and AB were responsible for histology assessments and consulting. JV and IK acquired patient data. KN, EM and AB performed laboratory assessments. KN and AP conducted imaging and image analysis. KN performed statistical analysis. JV, KB, IK, AP and AK provided a critical review. KN, EM and AB drafted the manuscript. All authors reviewed and approved the final manuscript.

## Supporting information


Supplementary materials and methods
**Figure S1.** Image analysis workflow
**Figure S2.** Creation of virtual stained, fluorescence‐like whole core images
**Figure S3.** Associations between ductal involvement, MUC5, MUC6, CDX2 profiles, and the expression of NLRP3 inflammasome proteins
**Figure S4.** Associations between dysplasia and the expression of NLRP3 inflammasome proteins in IPMN cases
**Figure S5.** NLRP3‐associated protein expression patterns and invasiveness in different subtypes of IPMN
**Figure S6.** Relationship between pathological prognostic factors, inflammasome protein expression, and IL‐18/IL‐18BP ratio in PDAC cases
**Figure S7.** The prognostic role of pathological factors in IPMN and PDAC
**Table S1.** Staining protocol of the NLRP3 inflammasome proteins on the TMA samples
**Table S2.** Staining protocol of the mucins and CDX2 protein on a serial section
**Table S3.** List of reagents used in immunohistochemical staining
**Table S4.** List of antibodies used in immunohistochemical staining

## Data Availability

The datasets generated and analyzed during the current study are available from the corresponding author(s) upon reasonable request.
