# Supplementary material for: Subtype‐associated complexity and prognostic significance of the NLRP3 inflammasome landscape in pancreatic neoplasms
Source: J Pathol Clin Res. 2025 Feb 19;11(2):e70019. doi: 10.1002/2056-4538.70019 (PMC11837281; doi:10.1002/2056-4538.70019)
Supplement: Supplementary file 1 — Supplementary materials and methods Figure S1. Image analysis workflow Figure S2. Creation of virtual stained, fluorescence‐like whole core images Figure S3. Associations between ductal involvement, MUC5, MUC6, CDX2 profiles, and the expression of NLRP3 inflammasome proteins Figure S4. Associations between dysplasia and the expression of NLRP3 inflammasome proteins in IPMN cases Figure S5. NLRP3‐associated protein expression patterns and invasiveness in different subtypes of IPMN Figure S6. Relationship between pathological prognostic factors, inflammasome protein expression, and IL‐18/IL‐18BP ratio in PDAC cases Figure S7. The prognostic role of pathological factors in IPMN and PDAC Table S1. Staining protocol of the NLRP3 inflammasome proteins on the TMA samples Table S2. Staining protocol of the mucins and CDX2 protein on a serial section Table S3. List of reagents used in immunohistochemical staining Table S4. List of antibodies used in immunohistochemical staining [file CJP2-11-e70019-s001.pdf]

# Subtype-associated complexity and prognostic significance of the NLRP3 inflammasome landscape in pancreatic neoplasms

K Nemeth *et al*, *J Pathol Clin Res*, https://doi.org/10.1002/2056-4538.70019

## Supplementary Material

### Table of contents

**Supplementary materials and methods** .......... 2

Multiplex immunohistochemistry .......... 2

Immunohistochemistry staining controls .......... 3

Image analysis .......... 10

**Supplementary figures** .......... 10

Figure S1 – Image analysis workflow .......... 11

Figure S2 – Creation of virtual stained, fluorescence-like whole core images .......... 12

Figure S3 - Associations between ductal involvement, MUC5, MUC6, CDX2 profiles, and the expression of NLRP3 inflammasome proteins .......... 13

Figure S4 - Associations between dysplasia and the expression of NLRP3 inflammasome proteins in IPMN cases .......... 14

Figure S5 - NLRP3-associated protein expression patterns and invasiveness in different subtypes of IPMN .......... 15

Figure S6 - Relationship between pathological prognostic factors, inflammasome protein expressions, and IL-18/IL-18BP ratio in PDAC cases .......... 16

Figure S7 - The prognostic role of pathological factors in IPMN and PDAC .......... 17

**Supplementary tables** .......... 18

Table S1- Staining protocol of the NLRP3 inflammasome proteins on the TMA samples .......... 18

Table S2- Staining protocol of the mucins and CDX2 protein on a serial section .......... 19

Table S3- List of reagents used in immunohistochemical staining .......... 20

Table S4- List of antibodies used in immunohistochemical staining .......... 21

**References** .......... 22

# Supplementary materials and methods

## Multiplex immunohistochemistry

The Multiplexed Immunohistochemical Consecutive Staining on Single Slide (MICSSS) technique was performed [1]. The slides were deparaffinized in xylene and rehydrated in a graded alcohol series. Heat-induced antigen retrieval was performed (30 minutes at 96 °C, at pH 6 or 9), and then slides were washed in Tris-buffered saline (TBS, 3X5 minutes). Endogenous peroxidase quenching was performed by 3% $H_2O_2$ for 15 minutes. Slides were washed in TBS (2x5 minutes), and then 5% milk-TBS was used for protein blocking (30 minutes). Primary antibodies were incubated for 60 minutes at room temperature, then slides were washed in 0.04% Tris-buffered saline with Tween 20 (TBST, 3x5 minutes). Host-specific secondary antibodies were added (60 minutes incubation, room temperature). Subsequently, slides were washed in TBST (5 minutes), and treated with peroxidase substrate (ImmPACT AMEC Red Substrate Kit, Vector Laboratories) (10 minutes, room temperature). After staining, the samples were washed in distilled water for 5 minutes and counterstaining was performed with hematoxylin (1 minute, room temperature). The hematoxylin labeling was followed by a 2-minute tap water and a distilled water wash (2X30 seconds). Coverslips were mounted using glycerol. After scanning at 20x magnification (Pannoramic RX1000 Slide Scanner, 3DHistech, Budapest, Hungary), the slides were washed with distilled water for 5 minutes, then the staining was removed with graded ethanol series. Then a new round of heat-induced antigen retrieval (15 minutes at 96°C, at pH 6 or 9) and after that a host-specific fragment antibody (fAb) incubation for 60 minutes and TBST washing for 2X5 minutes was included to enable safe consecutive staining. To check the effectiveness of fAb blockade, after each staining round, the blocked positive control was reincubated with the secondary antibody and AMEC. Details of the staining protocol, including the reagents and antibodies used, and the parameters of the scanning can be found in Tables S1, S2, S3 and S4.

## Immunohistochemistry staining controls

NLRP3

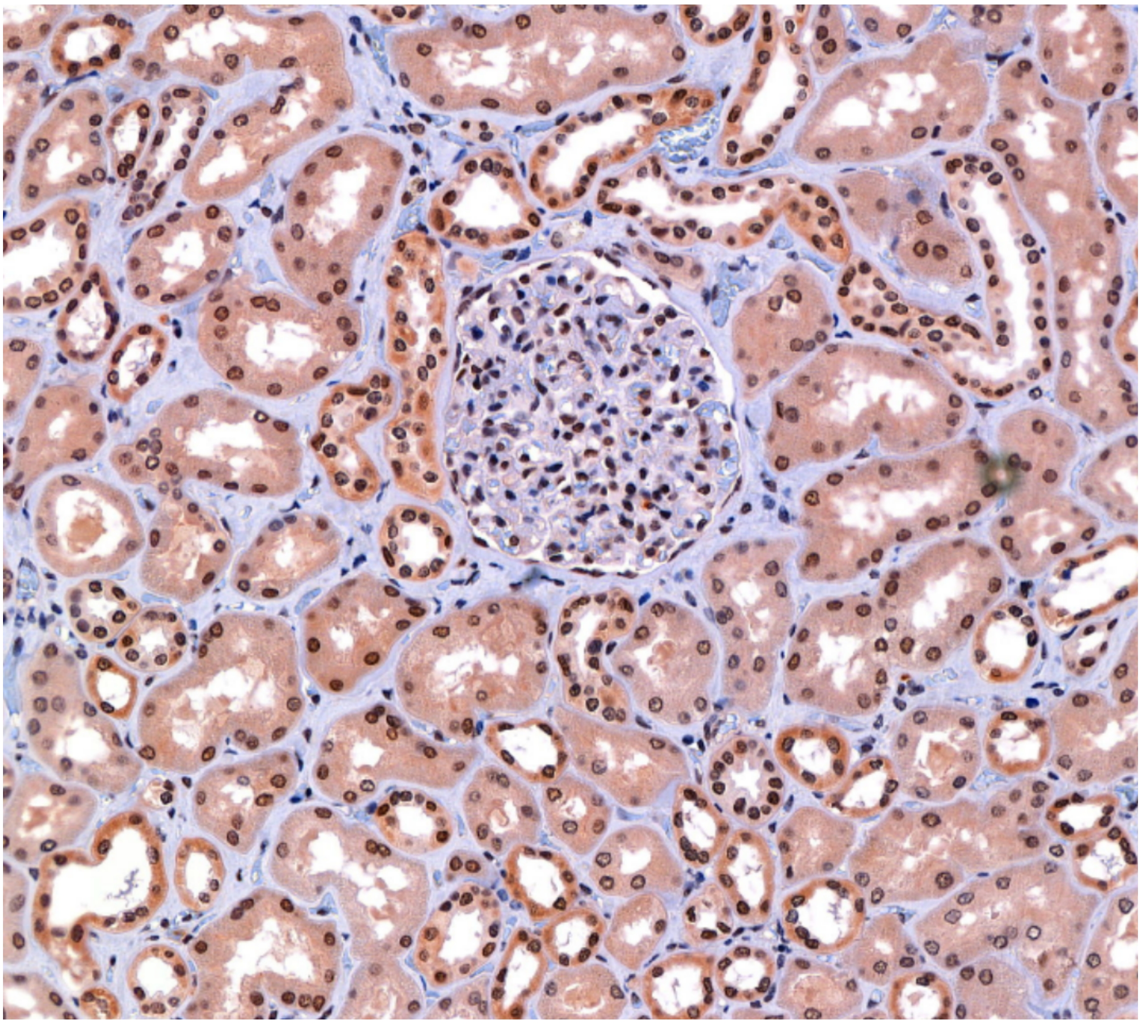

Kidney stained with NLRP3 antibody (dilution: 1:100, NBP2-124466, Novus Biologicals). Cells in tubuli show weak to medium intensity staining, while cells in glomeruli show no detectable reaction.
Secondary antibody: Rabbit IgG VisUCyte HRP Polymer Antibody (RTU, VC003-025, R&D Systems), Chromogen: ImmPACT AMEC Red Substrate Kit, Peroxidase (HRP) (SK-4285, Vector Laboratories)

Staging patterns are consistent with the data reported in The Human Protein atlas [2].

ASC

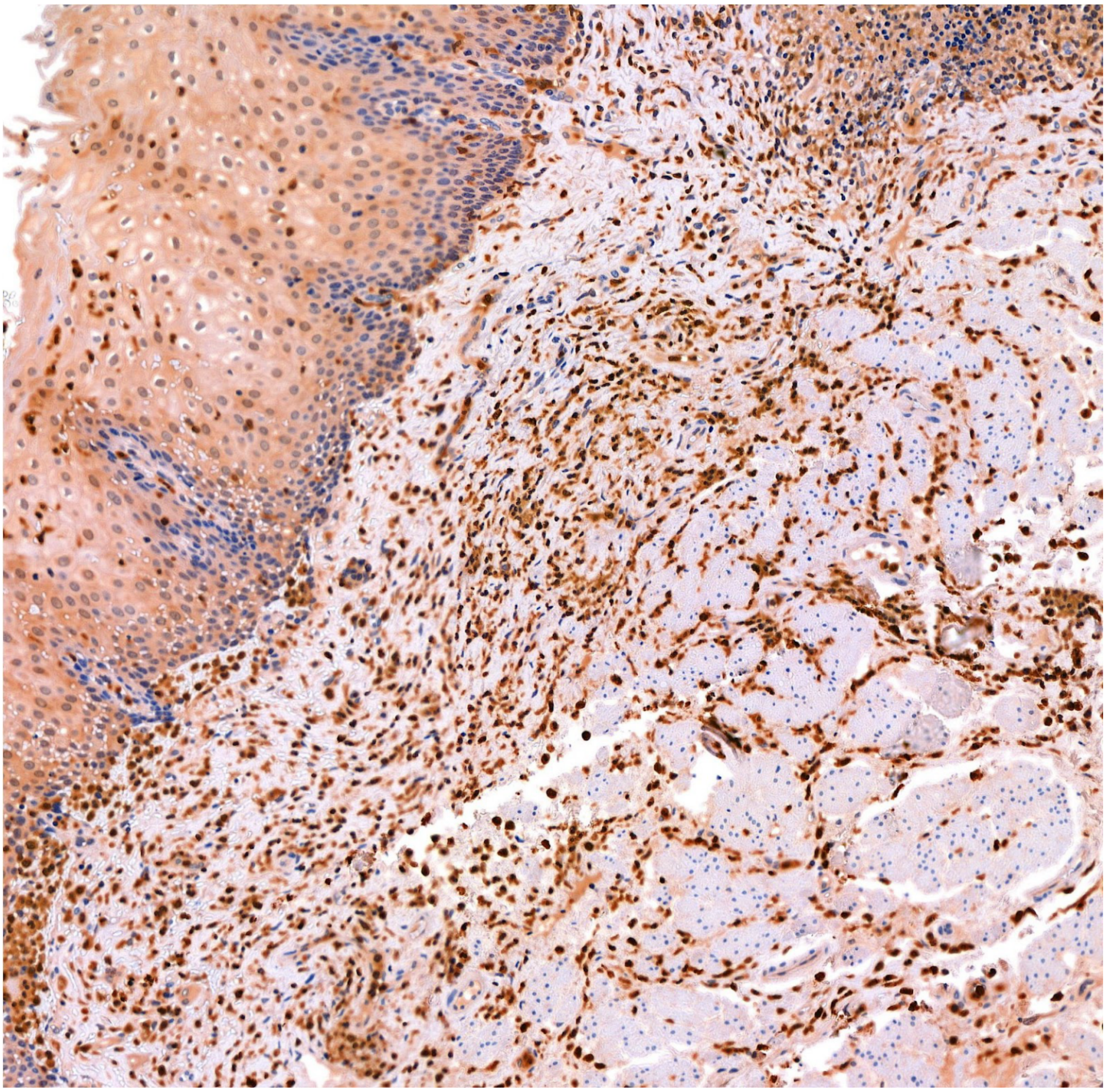

Oesophagus sample stained with anti-ASC antibody (ASC/TMS1 (E1E3I) Rabbit mAb (1:100, 13833S, Cell Signaling Technology). Lymphocytes and squamous epithelium cells (positive control) have a distinctive staining pattern, while smooth muscle cells, and epithelial basal cells (negative control) show no reactivity.

Secondary antibody: Rabbit IgG VisUCyte HRP Polymer Antibody (RTU, VC003-025, R&D Systems), Chromogen: ImmPACT AMEC Red Substrate Kit, Peroxidase (HRP) (SK-4285, Vector Laboratories)

Staging patterns are consistent with the data reported in The Human Protein atlas [3].

Caspase-1

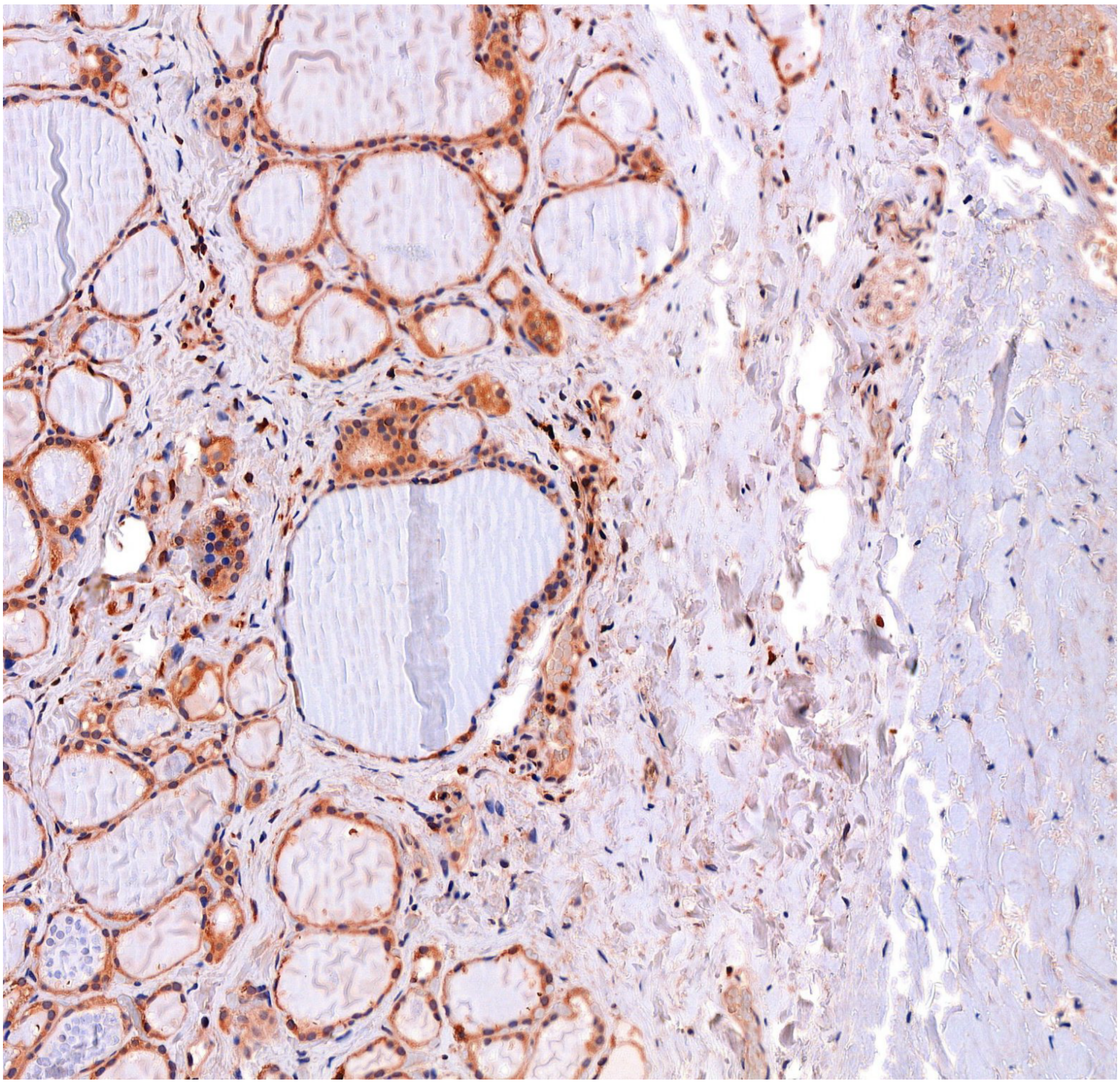

Thyroid gland stained with anti Caspase-1 antibody, (1:100, NB100-56565, Novus Biologicals). Tyreocytes and lymphocytes have a strong, distinctive staining (positive control), while fibroblasts and fibrocytes show no reactivity (negative control).

Secondary antibody: Mouse IgG VisUCyte HRP Polymer Antibody (RTU, VC001-025, R&D Systems), Chromogen: ImmPACT AMEC Red Substrate Kit, Peroxidase (HRP) (SK-4285, Vector Laboratories)

Staining pattern are consistent with the expression data reported in The Human Protein Atlas [4].

IL-1B

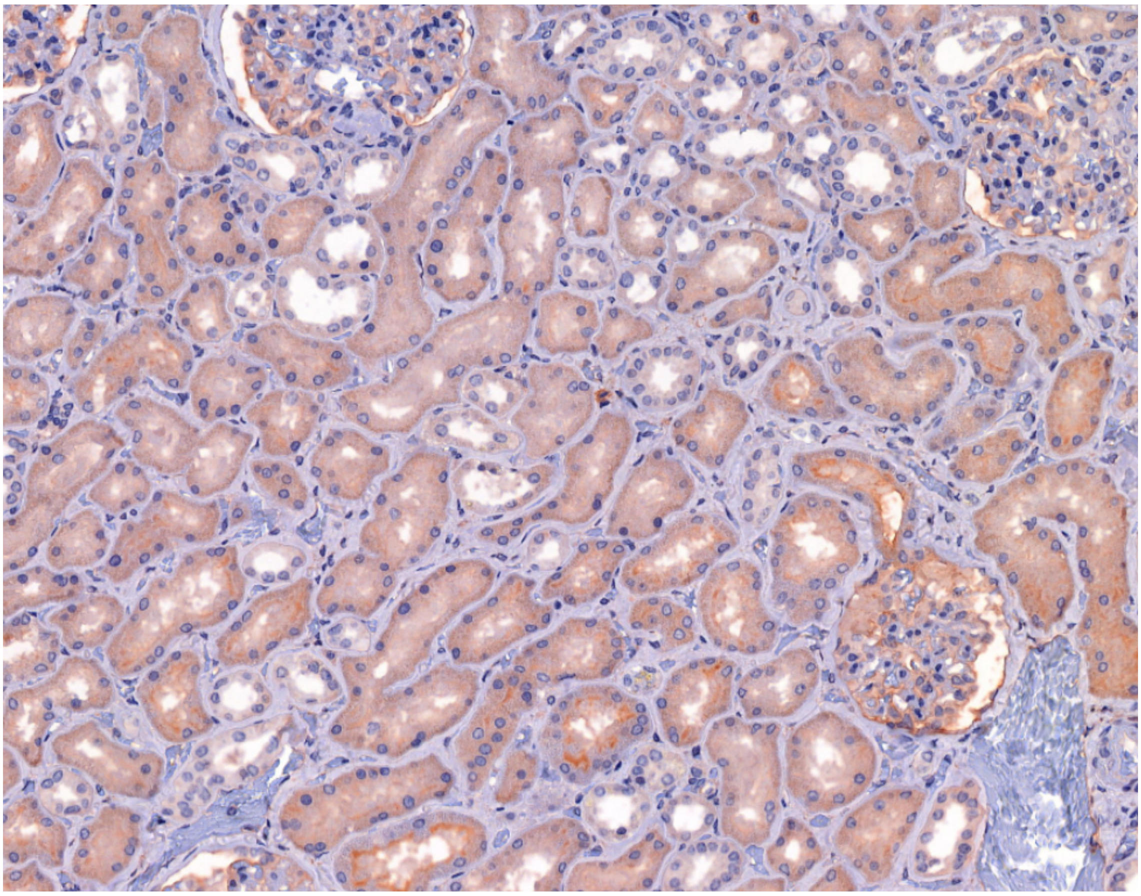

Kidney stained with anti-interleukin 1 beta antibody (1:100, ab9722, Abcam): Tubular epithelium shows weak medium strength staining (positive control), while endothelial cells showed no reaction (negative control).

Secondary antibody: Rabbit IgG VisUCyte HRP Polymer Antibody (RTU, VC003-025, R&D Systems), Chromogen: ImmPACT AMEC Red Substrate Kit, Peroxidase (HRP) (SK-4285, Vector Laboratories)

The findings are highly consistent with previously published data [5].

IL-18

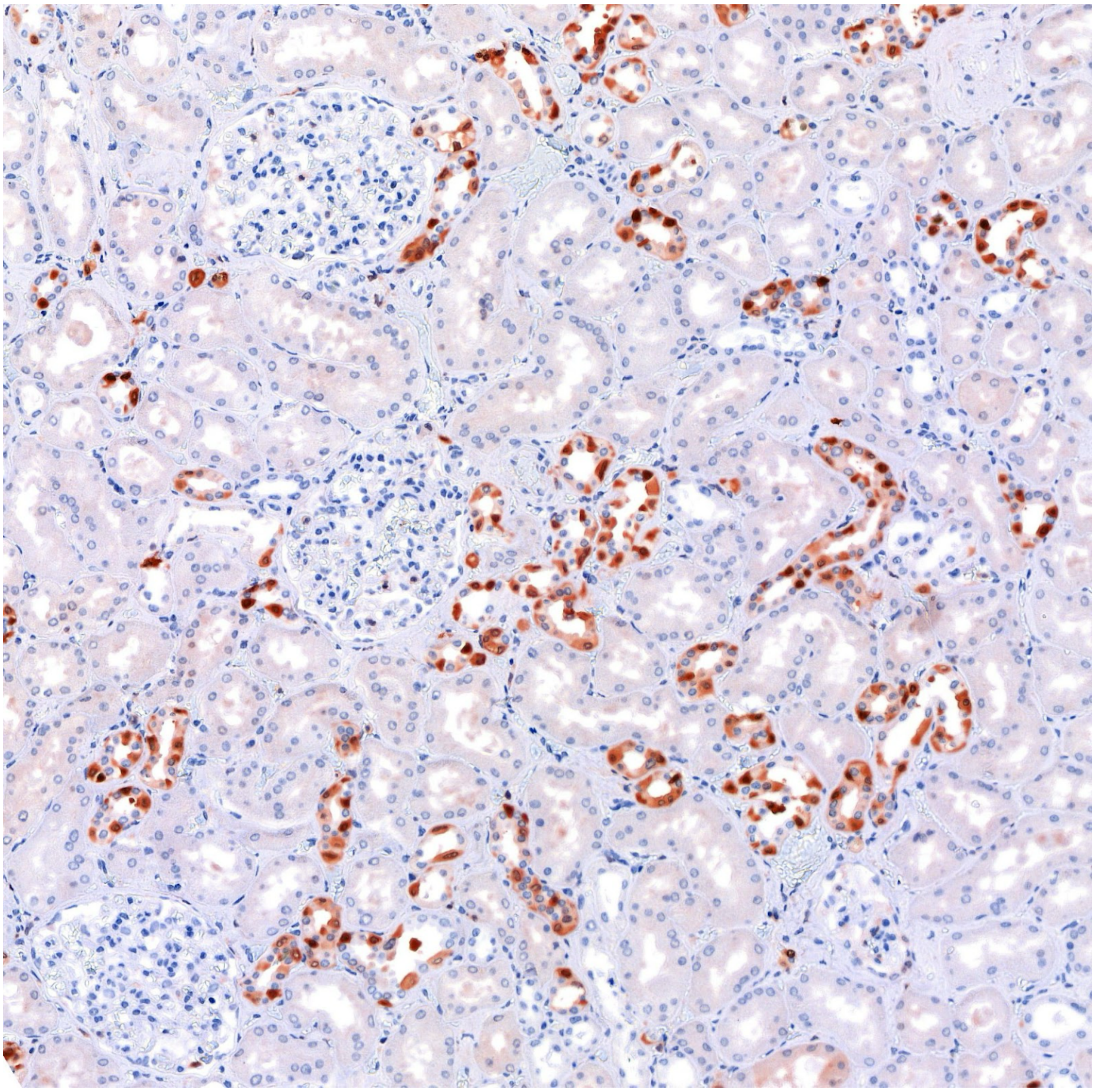

Kidney stained with anti-interleukin 18 antibody (1:250, ab243091, Abcam): While collecting ducts, distal and proximal tubules have variable intensity staining pattern (positive control), glomeruli has no reactivity with the antibody (negative control). Secondary antibody: Rabbit IgG VisUCyte HRP Polymer Antibody (RTU, VC003-025, R&D Systems), Chromogen: ImmPACT AMEC Red Substrate Kit, Peroxidase (HRP) (SK-4285, Vector Laboratories)

Staining patterns are consistent with that shown in The Human Protein Atlas [6].

IL-1RA

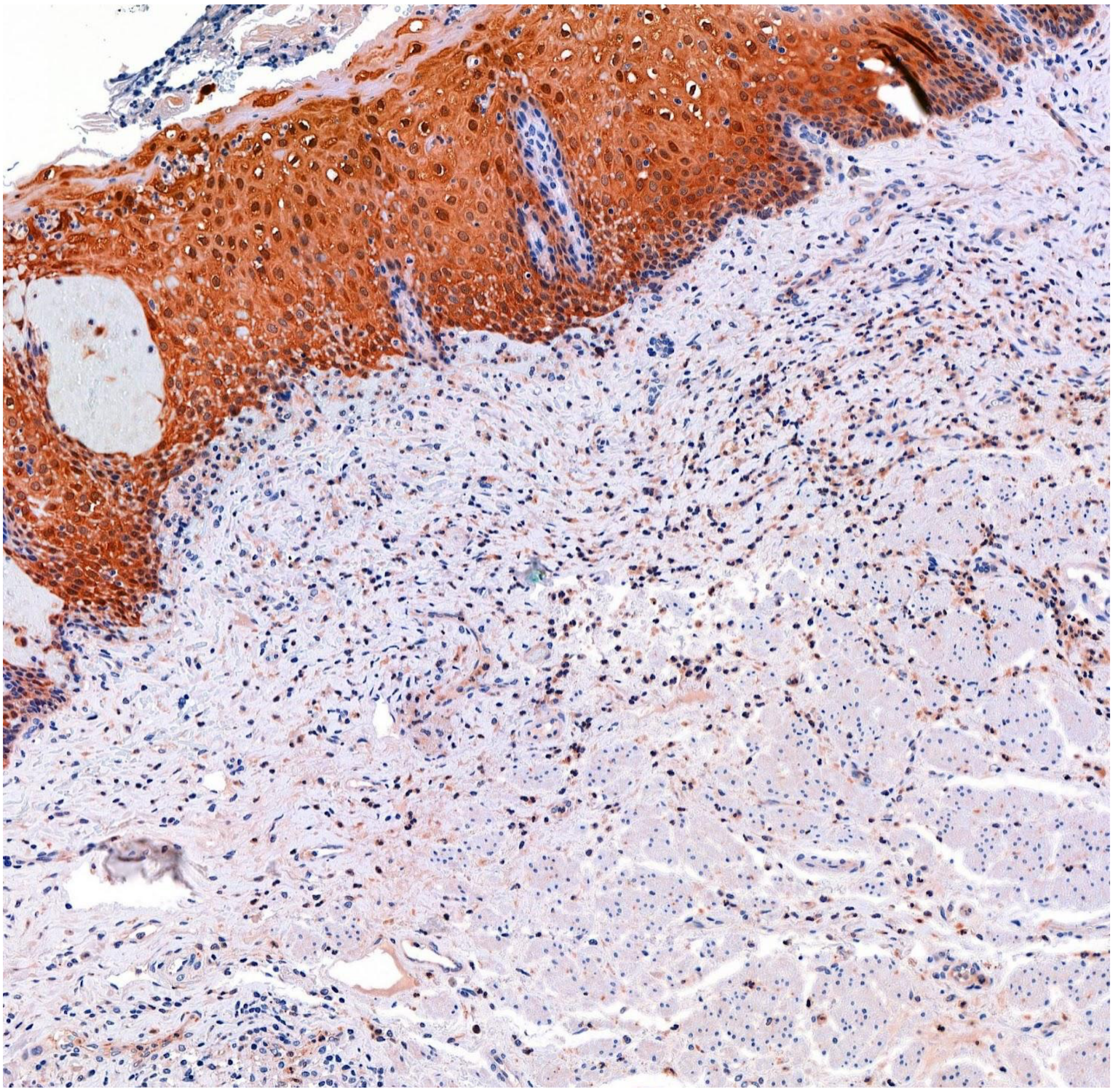

Oesophagus sample stained with anti-IL1RA antibody (1:100, ab124962, Abcam): Squamous cell epithelium and lymphocytes have distinctive strong staining (positive control), while fibrocytes and smooth muscle cells do not show reactivity (negative control).

Secondary antibody: Rabbit IgG VisUCyte HRP Polymer Antibody (RTU, VC003-025, R&D Systems), Chromogen: ImmPACT AMEC Red Substrate Kit, Peroxidase (HRP) (SK-4285, Vector Laboratories)

The seen expression patterns were similar that of those published in The Human Protein Atlas [7].

IL-18BP

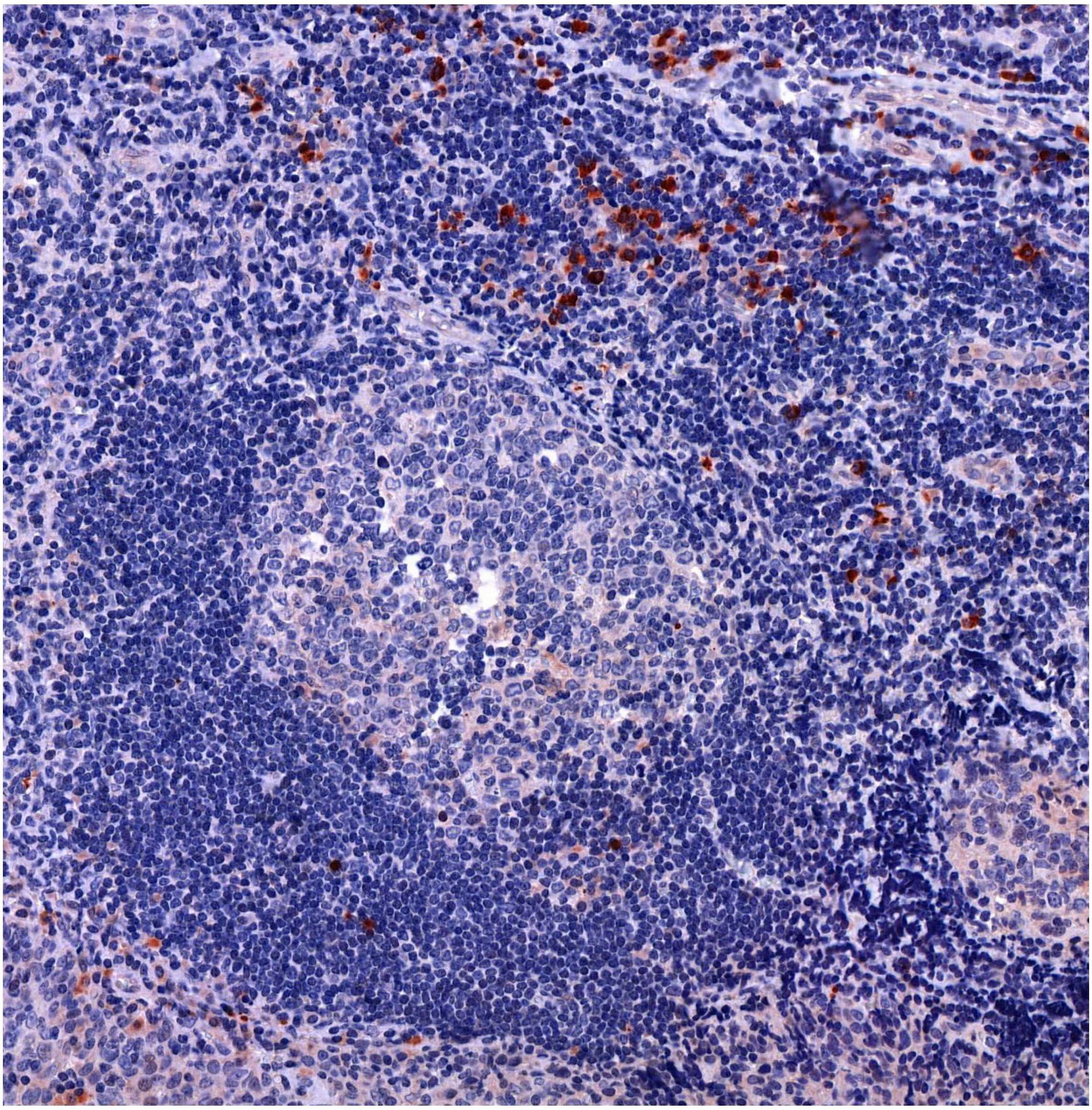

Tonsil stained with anti-interleukin 18 Binding Protein antibody (1:250, NBP2-38481, Novus Biologicals): Squamous epithelium dendritic cells and tingible macrophages show distinctive positive staining (Positive control), while marginal zone cells show no reactivity (negative control).

Secondary antibody: Rabbit IgG VisUCyte HRP Polymer Antibody (RTU, VC003-025, R&D Systems), Chromogen: ImmPACT AMEC Red Substrate Kit, Peroxidase (HRP) (SK-4285, Vector Laboratories)

Staining patterns are consistent with that shown in The Human Protein Atlas [8].

## Image analysis

Slides were imported into QuPath 0.4.0 [9]. For chromogen channel deconvolution, the color vector parameters for each stain were determined using the "estimate stain vectors" feature. Each core was annotated with the help of the “TMA dearrayer” feature and then exported as an RGB image in ”.tiff” format. After exporting, we generated a stack of images belonging to a core and aligned them using the TrakEM2 plugin in ImageJ [10]. To identify tumor cells, images stained with PanCK were segmented using the Ilastik machine-learning-based segmentation toolkit [11]. Subsequently, the initial segmentation masks were validated and manually corrected by a pathologist specializing in hepato-pancreato-biliary pathology. After image alignment and tumor cell segmentation, further analysis was performed in ImageJ [12]. The images were deconvoluted using previously estimated staining-specific color vector parameters, and the AMEC channel images were then extracted in 8-bit. Following that, the segmentation mask for a specific core was applied to the 7 AMEC channel images corresponding to the same core (each protein marker comes with one image), cutting out the tumor area. Next, intensity histograms for each stained image were created based on this tumor area. The histogram data were used to determine the Averaged Threshold Measure (ATM) score, which score system based on the mathematical formula described by Choudhury et al. [13]. For non-invasive IPMN and PDAC cases, the ATM score was calculated as the sum of intensity histograms from all associated cores. For invasive IPMN cases, ATM scores were calculated separately for cores containing dysplastic and invasive components. The ATM scores derived from the invasive component cores were used for subsequent analyses. To calculate the ratio of the effector and the antagonist cytokines, the raw ATM score data were standardized by dividing with the molecule-specific mean of each protein and creating a ratio from the resulting relative expressions. The workflow of the image analysis and the mathematical formula of cytokine ratio calculation are described in Supplementary Figure S1.

## Supplementary figures

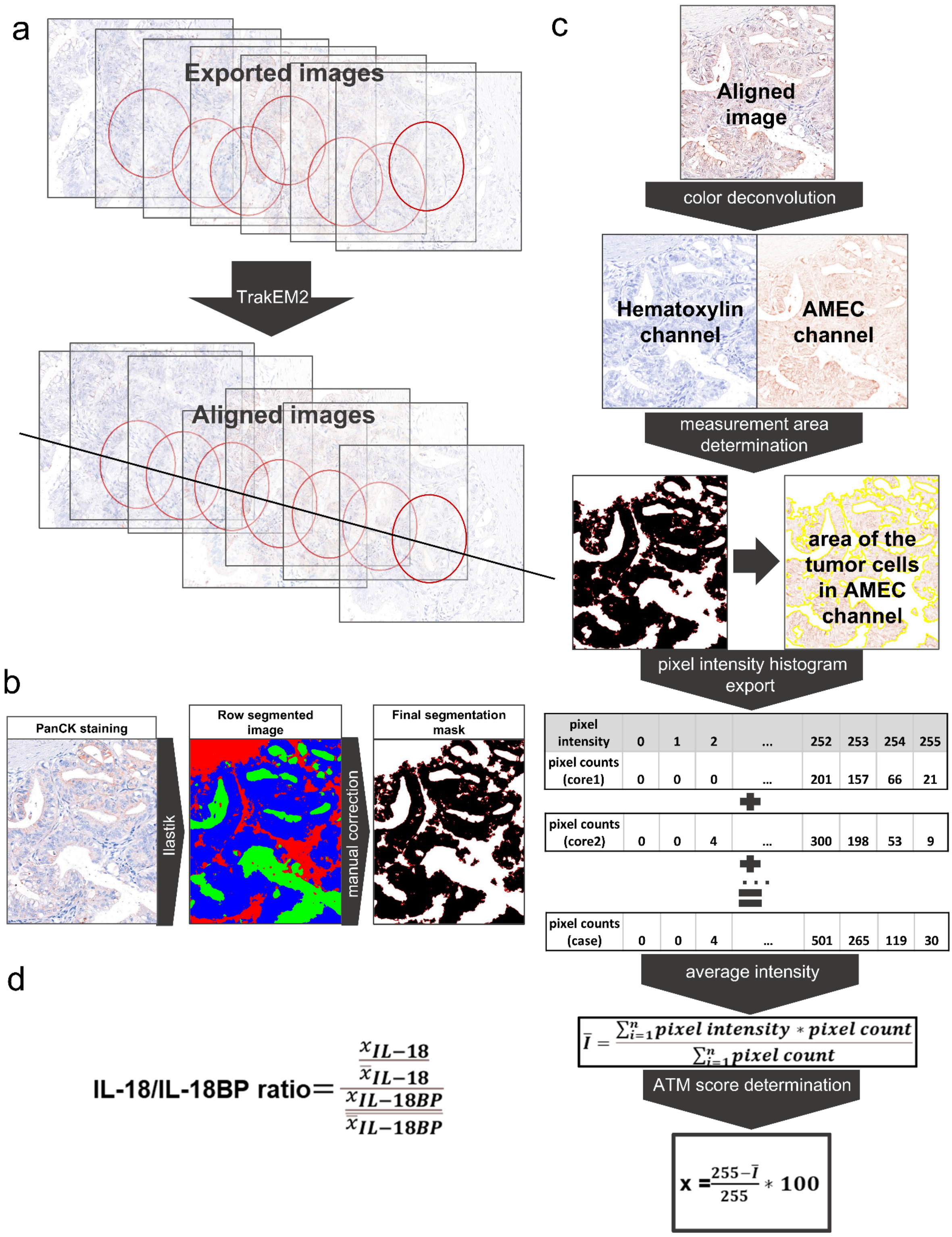


**Figure S1. Image analysis workflow.**
**(a)** Automated image alignment with the help of TrakEM2, an ImageJ plugin. **(b)** Determination of the tumor cell area. **(c)** ATM score determination workflow. **(d)** Calculation of cytokine ratios. Symbols: $\overline{I} =$ average intensity; x= ATM score of a case; $\overline{x} =$ average ATM score of a protein, based on all the cases

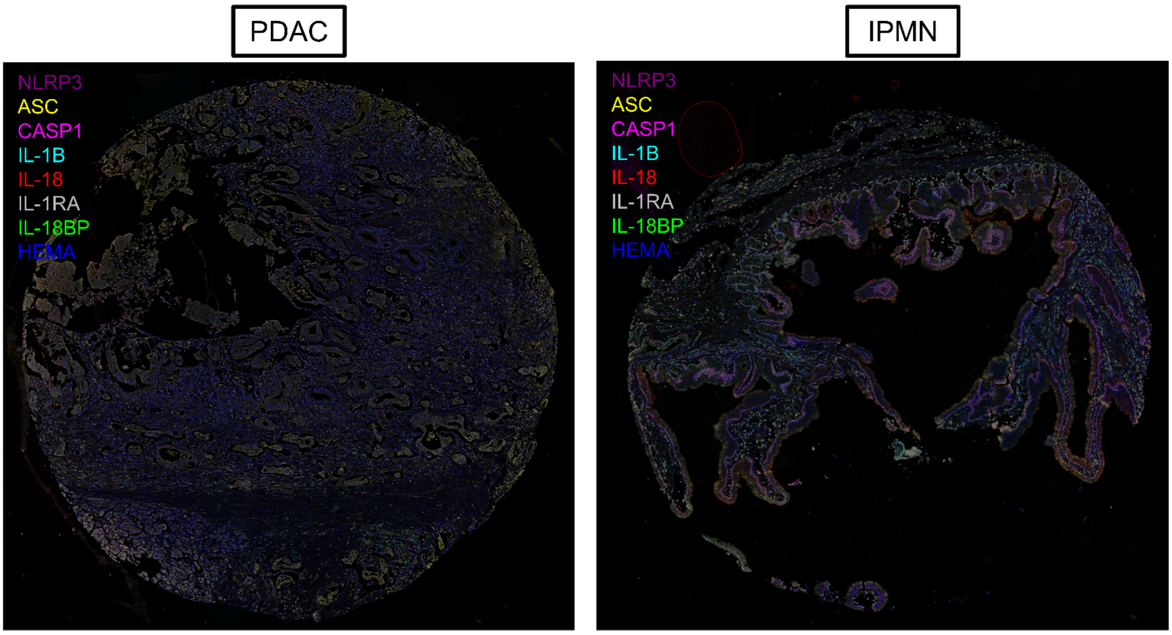


**Figure S2. Creation of virtual stained, fluorescence-like whole core images.**
Color deconvolution is performed separately on images of individual NLRP3 inflammasome proteins labeled with AMEC and hematoxylin, based on previously determined color vectors. Then, an arbitrary Look-Up Table (LUT) is assigned to the 8-bit image of the AMEC channel, artificially recoloring the image. A composite image is then created from these recolored 8-bit images.

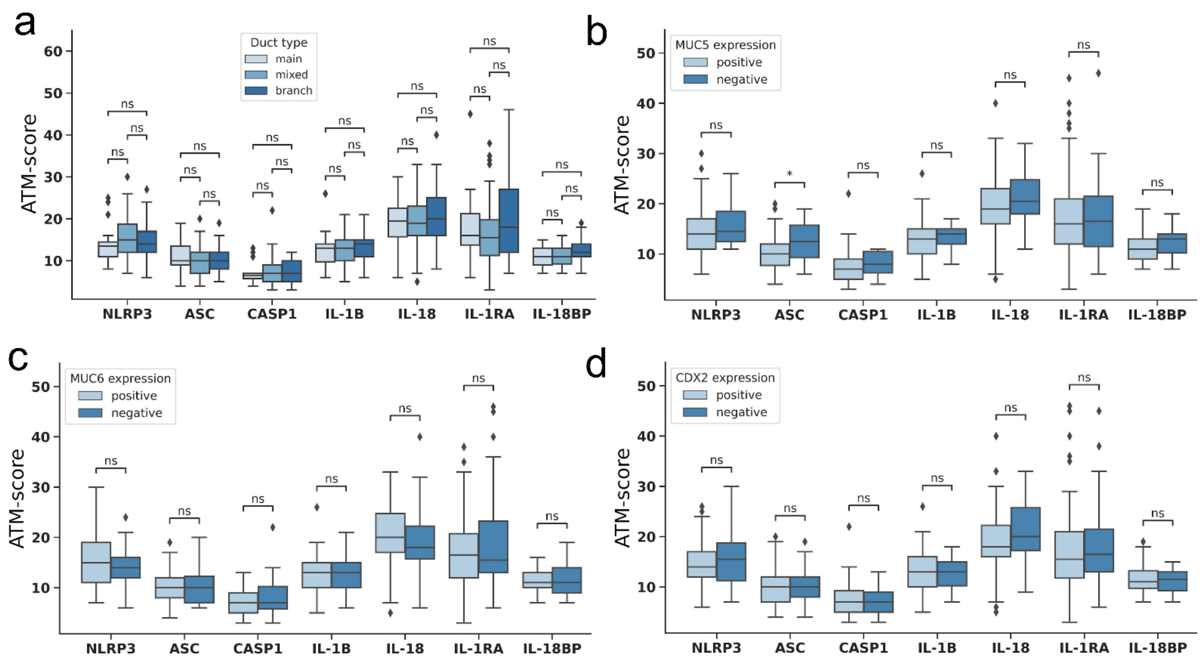


**Figure S3. Associations between ductal involvement, MUC5, MUC6, CDX2 profiles, and the expression of NLRP3 inflammasome proteins.**
(a–d) Association between the ATM scores of inflammasome proteins and ductal involvement (a) ($N_{main}$=17, $N_{mixed}$=60, $N_{branch}$=25), MUC5 expression (b) ($N_{positive}$=86, $N_{negative}$=14), MUC6 expression (c) ($N_{positive}$=58, $N_{negative}$=42), and CDX2 expression (d) ($N_{positive}$=62, $N_{negative}$=42). Lines represent medians, boxes represent interquartile ranges, and whiskers extend 1.5 times the interquartile ranges. Points represent outliers. Statistical analysis: Wilcoxon test, markings: ns p>0.05, *p<0.05, **p<0.01, ***p<0.001, ****p<0.0001. Abbreviations: CASP1, Caspase-1

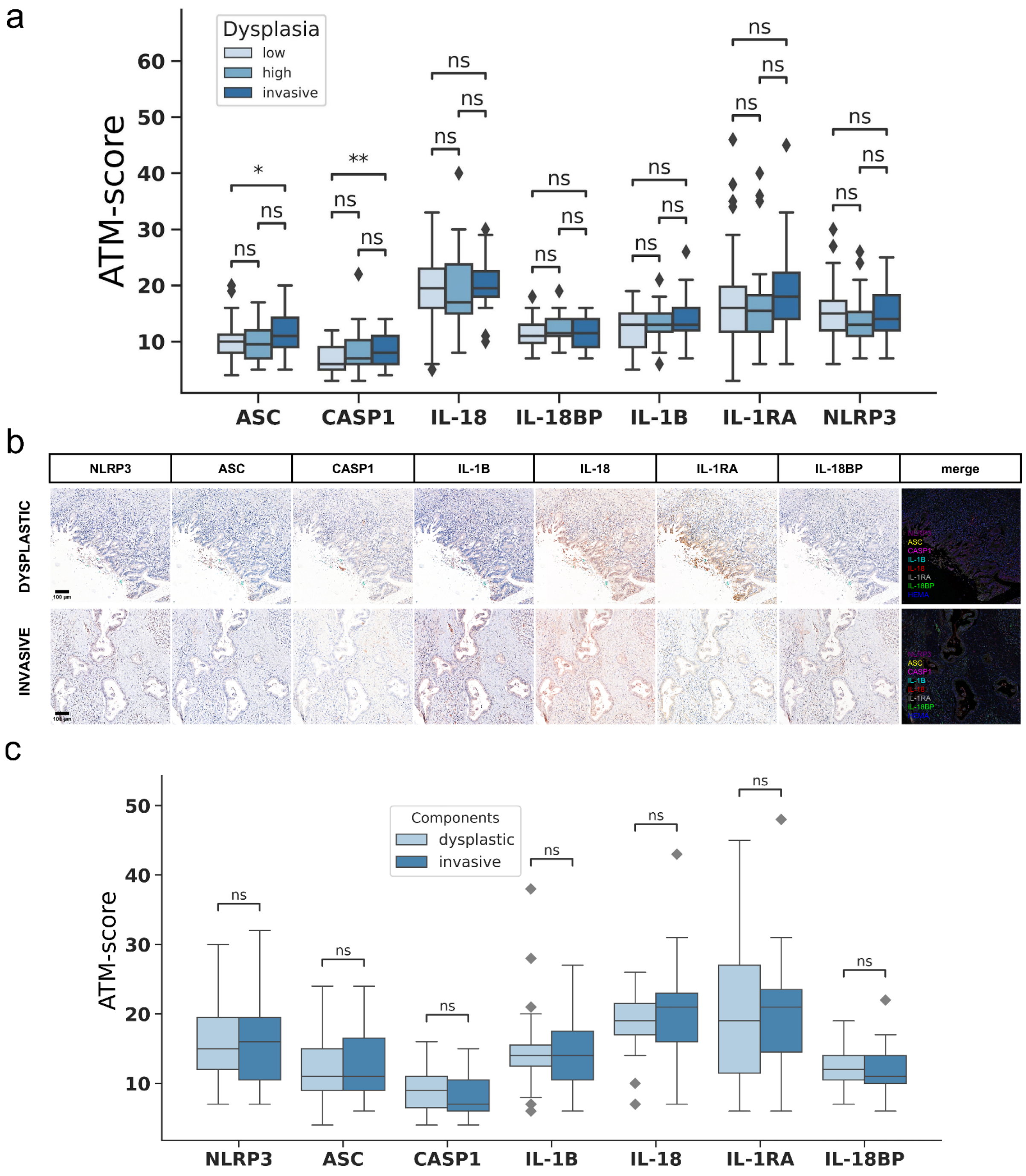


**Figure S4. Associations between dysplasia and the expression of NLRP3 inflammasome proteins in IPMN cases**

**(a)** Quantitative comparison of ATM scores based Figure S4. on dysplasia of IPMN cases ($N_{low}$=48, $N_{high}$=20, $N_{invasive}$=32). **(b)** Immunohistochemical staining patterns of dysplastic and invasive components of invasive IPMN cases based on representative images, showing AMEC and hematoxylin counterstaining and merged fluorescence-like images of all examined proteins (merge) (scale bar: 100 µm). **(c)** Quantitative comparison using ATM scores between the dysplastic and invasive components of invasive IPMN cases. Lines represent medians, boxes represent interquartile ranges, and whiskers extend 1.5 times the interquartile ranges. Points represent outliers. Statistical analysis: Wilcoxon test, markings: ns p>0.05, *p<0.05, **p<0.01, ***p<0.001, ****p<0.0001. Abbreviations: CASP1, Caspase-1

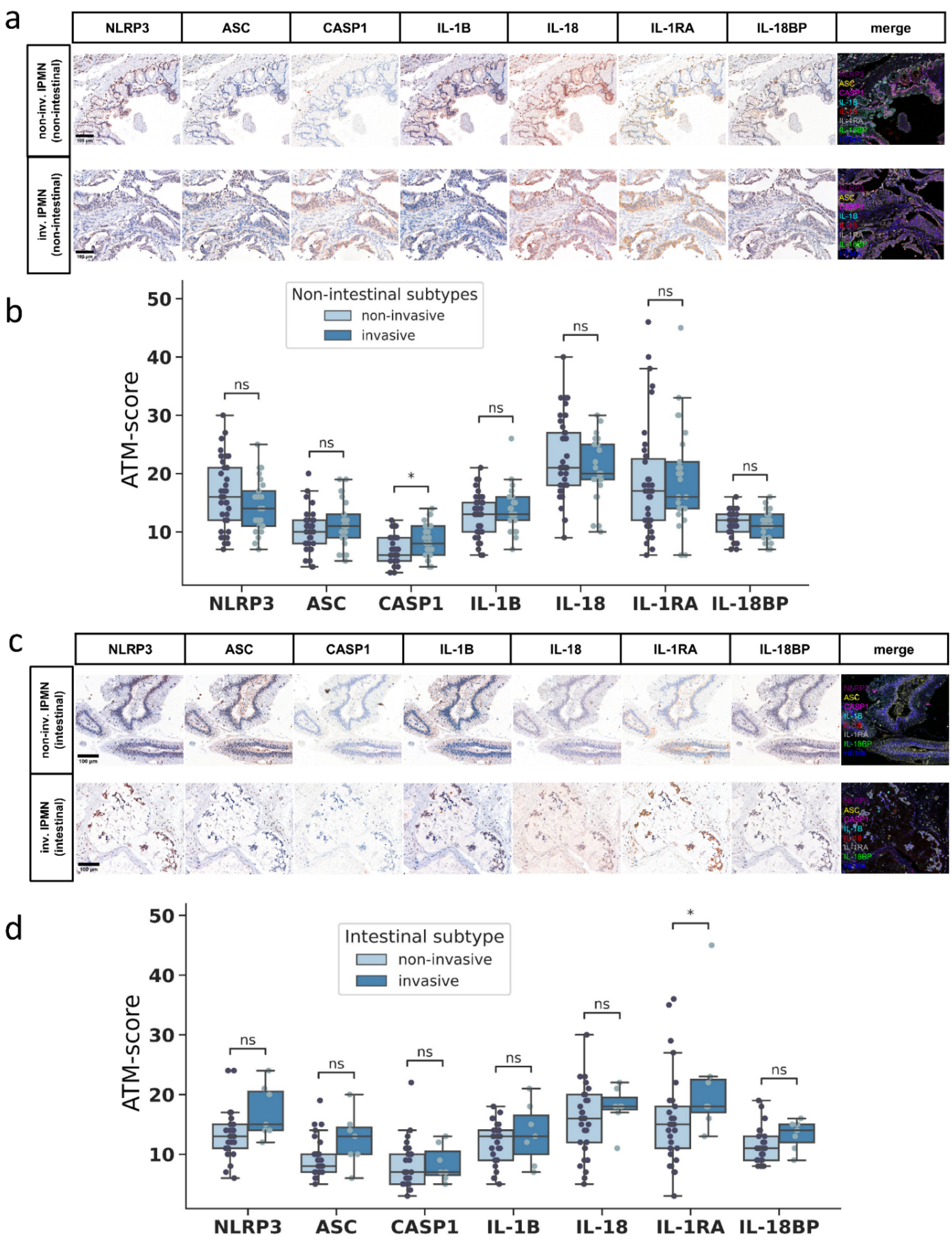


**Figure S5. NLRP3-associated protein expression patterns and invasiveness in different subtypes of IPMN**

**(a)** Immunohistochemical staining patterns of invasive and non-invasive, non-intestinal subtype IPMN cases based on representative images, showing AMEC and hematoxylin counterstaining and merged fluorescence-like images of all examined proteins (merge) (scale bar: 100 µm). **(b)** Quantitative comparison of ATM scores based on invasiveness in non-

intestinal IPMN cases ($N_{invasive}$=25, $N_{non-invasive}$=39). **(c)** Immunohistochemical staining patterns of invasive and non-invasive, intestinal subtype IPMN cases based on representative images, showing AMEC and hematoxylin counterstaining and merged fluorescence-like images of all examined proteins (merge) (scale bar: 100 μm). **(d)** Quantitative comparison of ATM scores based on invasiveness in intestinal IPMN cases ($N_{invasive}$=7, $N_{non-invasive}$=29). Lines represent medians, boxes represent interquartile ranges, and whiskers extend 1.5 times the interquartile ranges. Individual data points are also shown. Statistical analysis: Wilcoxon test, markings: ns: non-significant, p>0.05, *p<0.05, **p<0.01, ***p<0.001, ****p<0.0001. Abbreviations: non-inv., non-invasive; inv. , invasive

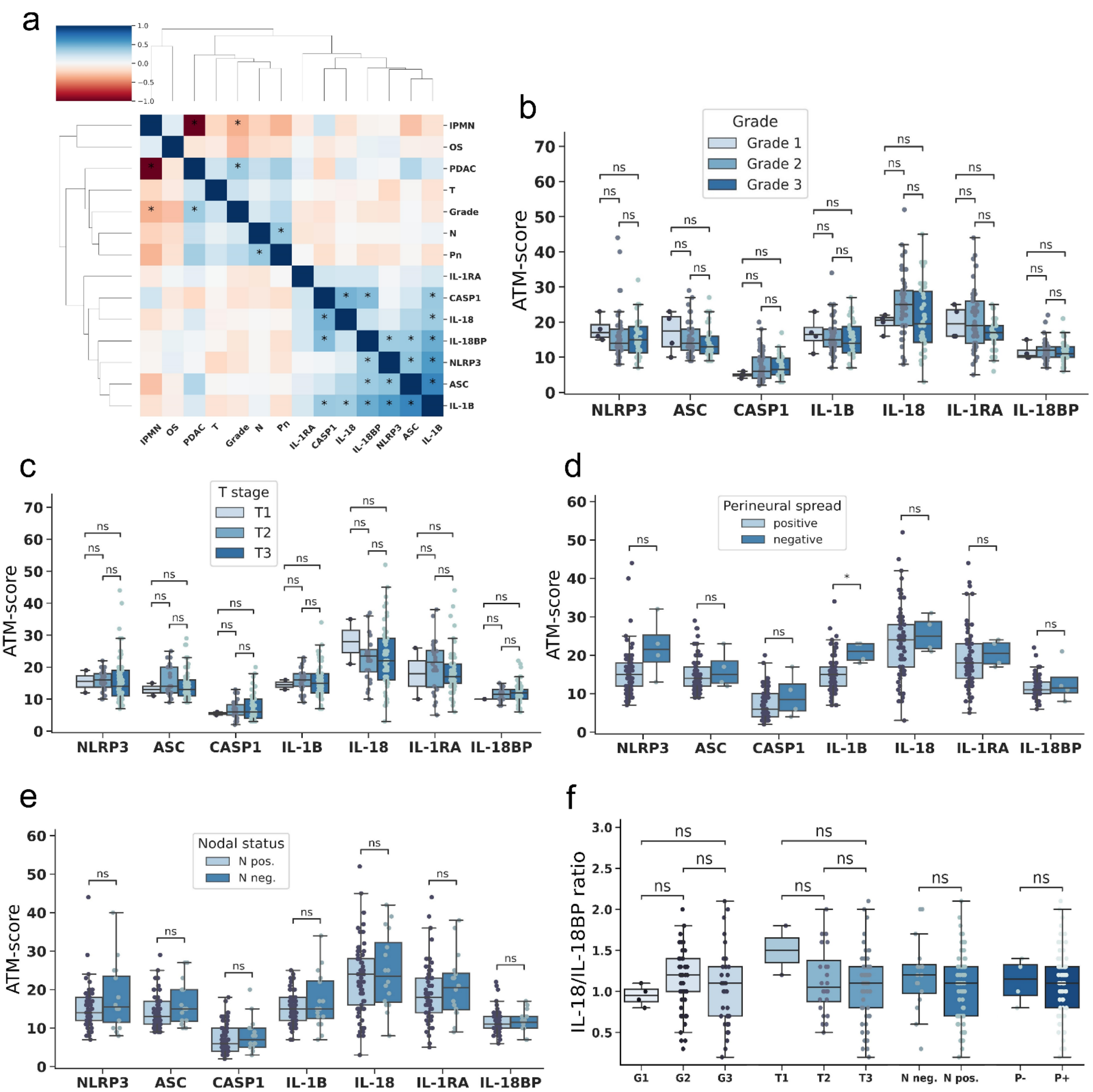

**Figure S6. Relationship between pathological prognostic factors, inflammasome protein expressions, and IL-18/IL-18BP ratio in PDAC cases**
**(a)** Heatmap demonstrates the correlation between examined proteins, pathological prognostic factors, and phenotype of the invasive IPMN and PDAC cases. The color scale represents the correlation coefficient, with blue indicating a positive correlation, and red indicating a negative correlation tendency. Statistical analysis: Spearman rank correlation analysis, markings: *p<0.05. **(b-e)** Association between inflammasome protein expression and grade **(b)** ($N_{G1}$=4, $N_{G2}$=49, $N_{G3}$=34), T stage **(c)** ($N_{T1}$=2, $N_{T2}$=24, $N_{T3}$=59), perineural spread **(d)** ($N_{positive}$=77, $N_{negative}$=6) and nodal status **(e)** ($N_{positive}$=69, $N_{negative}$=16). **(f)** Association between IL-18/IL-18BP ratio and pathological prognostic factors in PDAC cases. Lines represent medians, boxes represent interquartile ranges, and whiskers extend 1.5 times the interquartile ranges. Individual data points are also shown. Statistical analysis: Wilcoxon test, markings: ns: non-significant, p>0.05, *p<0.05, **p<0.01, ***p<0.001, ****p<0.0001.

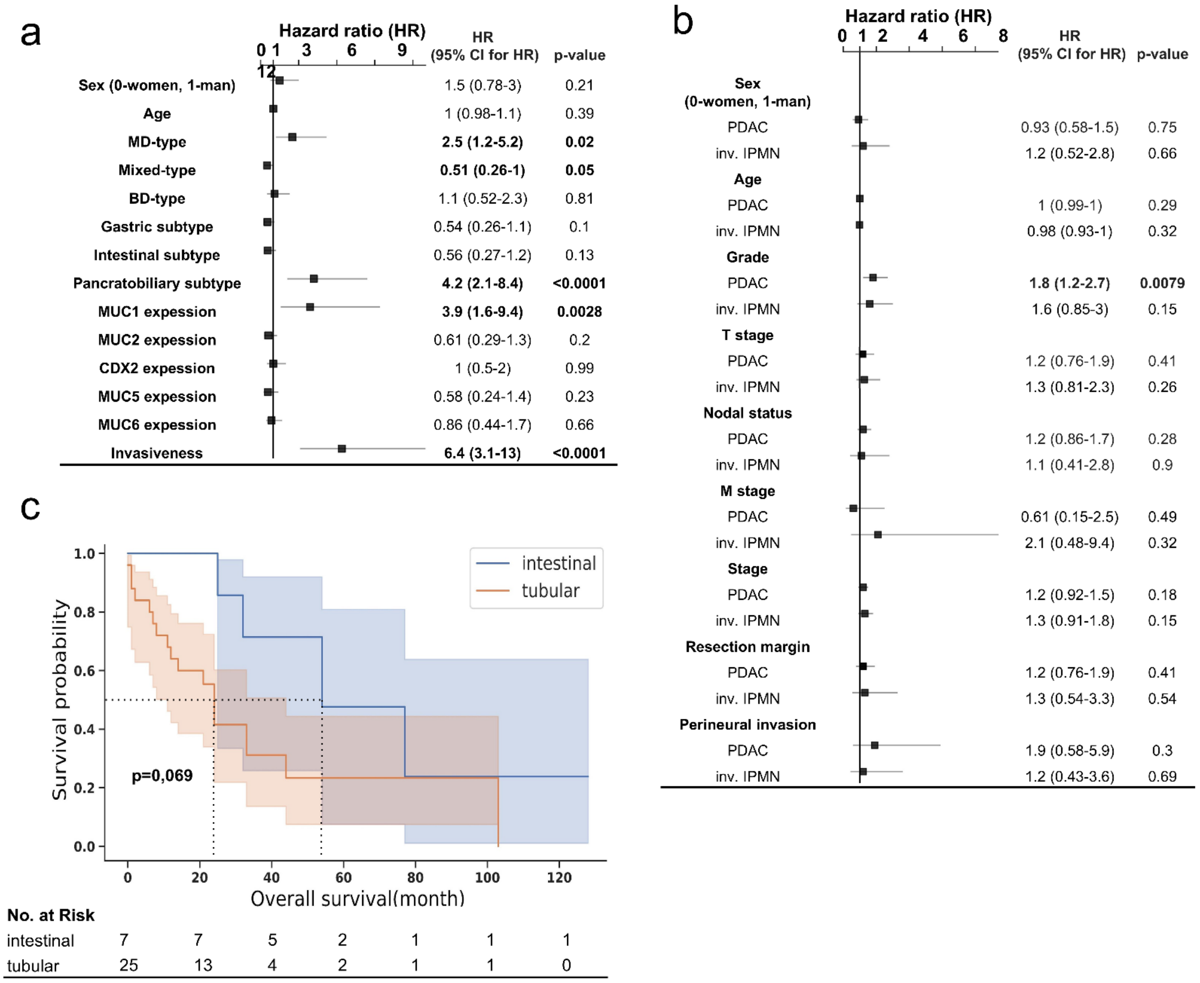


**Figure S7. The prognostic role of pathological factors in IPMN and PDAC**

**(a)** Impact of clinical and pathological factors on overall survival in IPMN cases, analyzed using the Cox proportional hazards model. **(b)** Comparison of the prognostic impact of clinical and pathological factors on overall survival in invasive IPMN and PDAC cases, analyzed using the Cox proportional hazards model. When p<0.05, the hazard ratio was considered significantly different. **(c)** Kaplan-Meier survival analysis of patients with intestinal and tubular type invasive IPMN. Median survival: 54 months (intestinal type) vs. 24 months (tubular type), p=0.069. Statistical significance was considered at p<0.05. Abbreviations: HR, hazard ratio; CI, confidence interval; inv., invasive;

## Supplementary tables

### Table S1. Staining protocol of the NLRP3 inflammasome proteins on the TMA samples

| | | IL-1RA | Caspase-1 | IL-18 | IL-1B | NLRP3 | ASC | IL-18BP | CK AE1+AE3 |
|---|---|---|---|---|---|---|---|---|---|
| 1/a. | **Deparaffinization** | ✓ | - | - | - | - | - | - | - |
| | **Xylol I.** | 20' | - | - | - | - | - | - | - |
| | **Xylol II.** | 20' | - | - | - | - | - | - | - |
| | **96% Ethanol** | 2' | - | - | - | - | - | - | - |
| | **70% Ethanol** | 15' | - | - | - | - | - | - | - |
| | **50% Ethanol** | 15' | - | - | - | - | - | - | - |
| | **Distilled water** | 5' | - | - | - | - | - | - | - |
| 1/b. | **Destaining** | - | ✓ | ✓ | ✓ | ✓ | ✓ | ✓ | ✓ |
| | **Coverslip elimination** | - | ✓ | ✓ | ✓ | ✓ | ✓ | ✓ | ✓ |
| | **Distilled water** | - | 5' | 5' | 5' | 5' | 5' | 5' | 5' |
| | **50% Ethanol** | - | 2' | 2' | 2' | 2' | 2' | 2' | 2' |
| | **1% HCl in 70% Ethanol** | - | 10' | 10' | 10' | 10' | 10' | 10' | 10' |
| | **96% Ethanol** | - | 5' | 5' | 5' | 5' | 5' | 5' | 5' |
| | **70% Ethanol** | - | 2' | 2' | 2' | 2' | 2' | 2' | 2' |
| | **50% Ethanol** | - | 2' | 2' | 2' | 2' | 2' | 2' | 2' |
| 2. | **Distilled water** | - | 5' | 5' | 5' | 5' | 5' | 5' | 5' |
| 3. | **Heat-Induced Epitope Retrieval ( 80 mL)** | 30' | 15' | 30' | 15' | 15' | 15' | 15' | 15' |
| | **pH** | 6 | 6 | 9 | 6 | 9 | 6 | 6 | 6 |
| 4. | **Cooling** | 30' | 10' | 30' | 10' | 10' | 10' | 10' | 10' |
| 5. | **1% TBS** | 2X5' | 2X5' | 2X5' | 2X5' | 2X5' | 2X5' | 2X5' | 2X5' |
| 6. | **Peroxidase blocking (3% H2O2)** | 30' | 10' | 10' | 10' | 10' | 10' | 10' | 10' |
| 7. | **1% TBS** | 30" | 30" | 30" | 30" | 30" | 30" | 30" | 30" |
| 8. | **Protein blocking (5% milk powder in 1% TBS)** | 30' | 30' | 30' | 30' | 30' | 30' | 30' | 30' |
| 9. | **1% TBS** | 30" | 30" | 30" | 30" | 30" | 30" | 30" | 30" |
| 10. | **fAb (1:40, 80 µL/slide)** | - | - | 60' | 60' | 60' | 60' | 60' | 60' |
| | **against** | - | - | Rb | Rb | Rb | Rb | Rb | Rb+Ms |
| | **host** | - | - | Dk | Dk | Dk | Dk | Dk | Dk |
| 11. | **TBS20 (0.04% Tween in 1% TBS)** | - | - | 3X5' | 3X5' | 3X5' | 3X5' | 3X5' | 3X5' |
| 12. | **Primary Antibody (1:100, room temperature, 80 µL/slide)** | 60' | 60' | 60' | 60' | 60' | 60' | 60' | 60' |
| | **against** | human IL-1RA | human Caspase-1 | human IL-18 | human IL-1B | human NLRP3 | human ASC | human IL-18BP | human CK AE1+AE3 |
| | **host** | Rb | Ms | Rb | Rb | Rb | Rb | Rb | Ms |
| 13. | **TBS20 (0.04% Tween in 1% TBS)** | 2X5' | 2X5' | 2X5' | 2X5' | 2X5' | 2X5' | 2X5' | 2X5' |
| 14. | **1% TBS** | 30" | 30" | 30" | 30" | 30" | 30" | 30" | 30" |
| 15. | **Secondary Antibody (ready-to-use, room temperature, 80 µL/slide)** | 30' | 30' | 30' | 30' | 30' | 30' | 30' | 30' |
| | **against** | Rb | Ms | Rb | Rb | Rb | Rb | Rb | Ms |
| 17. | **TBS20 (0.04% Tween in 1% TBS)** | 2X5' | 2X5' | 2X5' | 2X5' | 2X5' | 2X5' | 2X5' | 2X5' |
| 18. | **1% TBS** | 30" | 30" | 30" | 30" | 30" | 30" | 30" | 30" |
| 19. | **AMEC reagent** | 10' | 10' | 10' | 10’ | 10' | 10' | 10' | 10' |
| 20. | **Distilled water** | 5' | 5' | 5' | 5' | 5' | 5' | 5' | 5' |
| 21. | **Hematoxylin** | 1' | 1' | 1' | 1' | 1' | 1' | 1' | 1' |
| 22. | **Lukewarm water** | 2' | 2' | 2' | 2' | 2' | 2' | 2' | 2' |
| 23. | **Distilled water** | 2X30" | 2X30" | 2X30" | 2X30" | 2X30" | 2X30" | 2X30" | 2X30" |
| 24. | **Glycerol mounting** | ✓ | ✓ | ✓ | ✓ | ✓ | ✓ | ✓ | ✓ |
| 25. | **Covering (coverslip + rubber cement)** | ✓ | ✓ | ✓ | ✓ | ✓ | ✓ | ✓ | ✓ |
| 26. | **Scanning** | ✓ | ✓ | ✓ | ✓ | ✓ | ✓ | ✓ | ✓ |

## Table S2. Staining protocol of the mucins and CDX2 protein on a serial section

| | | MUC6 | MUC2 | CDX2 | MUC1 | MUC5 |
|---|---|---|---|---|---|---|
| 1/a. | **Deparaffinization** | ✓ | - | - | - | - |
| | **Xylol I.** | 20' | - | - | - | - |
| | **Xylol II.** | 20' | - | - | - | - |
| | **96% Ethanol** | 2' | - | - | - | - |
| | **70% Ethanol** | 15' | - | - | - | - |
| | **50% Ethanol** | 15' | - | - | - | - |
| | **Distilled water** | 5' | - | - | - | - |
| 1/b. | **Destaining** | - | ✓ | ✓ | ✓ | ✓ |
| | **Coverslip elimination** | - | ✓ | ✓ | ✓ | ✓ |
| | **Distilled water** | - | 5' | 5' | 5' | 5' |
| | **50% Ethanol** | - | 2' | 2' | 2' | 2' |
| | **1% HCl in 70% Ethanol** | - | 10' | 10' | 10' | 10' |
| | **96% Ethanol** | - | 5' | 5' | 5' | 5' |
| | **70% Ethanol** | - | 2' | 2' | 2' | 2' |
| | **50% Ethanol** | - | 2' | 2' | 2' | 2' |
| 2. | **Distilled water** | - | 5' | 5' | 5' | 5' |
| 3. | **Heat-Induced Epitope Retrieval ( 80 mL)** | 30' | 25' | 45' | 45' | 20' |
| | **pH** | 9 | 9 | 9 | 9 | 9 |
| 4. | **Cooling** | 30' | 10' | 30' | 10' | 10' |
| 5. | **1% TBS** | 2X5' | 2X5' | 2X5' | 2X5' | 2X5' |
| 6. | **Peroxidase blocking (3% H2O2)** | 30' | 10' | 10' | 10' | 10' |
| 7. | **1% TBS** | 30" | 30" | 30" | 30" | 30" |
| 8. | **Protein blocking (5% milk powder in 1% TBS)** | 30' | 30' | 30' | 30' | 30' |
| 9. | **1% TBS** | 30" | 30" | 30" | 30" | 30" |
| 10. | **Fab Antibody (1:40, 80 µL/slide)** | - | 60' | overnight | overnight | 60' |
| | **against** | - | Ms | Ms | Ms and Rb | Ms |
| | **host** | - | Dk | Dk | Dk | Dk |
| 11. | **TBS20 (0.04% Tween in 1% TBS)** | - | 3X5' | 3X5' | 3X5' | 3X5' |
| 12. | **Primary Antibody ( room temperature, 80 µL/slide)** | 60' | 60' | 60' | 60' | 60' |
| | **dilution** | 1:100 | 1:100 | 1:200 | 1:100 | 1:100 |
| | **against** | human MUC6 | human MUC2 | human CDX2 | human MUC1 | human MUC5 |
| | **host** | Ms | Ms | Rb | Ms | Ms |
| 13. | **TBS20 (0.04% Tween in 1% TBS)** | 2X5' | 2X5' | 2X5' | 2X5' | 2X5' |
| 14. | **1% TBS** | 30" | 30" | 30" | 30" | 30" |
| 15. | **Secondary Antibody (ready-to-use, room temperature, 80 µL/slide)** | 30' | 30' | 30' | 30' | 30' |
| | **against** | Ms | Ms | Rb | Ms | Ms |
| 17. | **TBS20 (0.04% Tween in 1% TBS)** | 2X5' | 2X5' | 2X5' | 2X5' | 2X5' |
| 18. | **1% TBS** | 30" | 30" | 30" | 30" | 30" |
| 19. | **AMEC reagent **** | 15' | 15' | 15' | 15’ | 15' |
| 20. | **Distilled water** | 5' | 5' | 5' | 5' | 5' |
| 21. | **Hematoxylin** | 1' | 1' | 1' | 1' | 1' |
| 22. | **Lukewarm water** | 2' | 2' | 2' | 2' | 2' |
| 23. | **Distilled water** | 2X30" | 2X30" | 2X30" | 2X30" | 2X30" |
| 24. | **Glycerol mounting** | ✓ | ✓ | ✓ | ✓ | ✓ |
| 25. | **Covering (coverslip + rubber cement)** | ✓ | ✓ | ✓ | ✓ | ✓ |
| 26. | **Scanning** | ✓ | ✓ | ✓ | ✓ | ✓ |

## Table S3. List of reagents used in immunohistochemical staining

| Name | Cat. No | Company |
|---|---|---|
| Xylene (mixture of isomers) | 20E114015 | VWR Chemicals |
| Ethanol absolute | 22C174009 | VWR Chemicals |
| Hydrochloric acid, 37% | BCBG0983V | Sigma-Aldrich |
| Epitope Retrieval Solution pH 9 (x10 Concentrate) | RE7119-CE | Leica Biosystems |
| Epitope Retrieval Solution pH 6 (x10 Concentrate) | RE7113-CE | Leica Biosystems |
| Hydrogen peroxide (H2O2) | 03650 | Molar Chemicals |
| Milk powder (low-fat milk powder) | 361834009 | Tutti Élelmiszeripari Kft. |
| Tween 20, 100% Nonionic Detergent | 1706531 | Bio-Rad Laboratories |
| TBS Powder | HY-K1026 | MedChemExpress |
| Mouse IgG VisUCyte HRP Polymer Antibody | VC001-025 | R&D Systems |
| Rabbit IgG VisUCyte HRP Polymer Antibody | VC003-025 | R&D Systems |
| AffiniPure Fab Fragment Donkey Anti-Rabbit IgG (H+L) | 711-007-003 | Jackson ImmunoResearch Laboratories |
| AffiniPure Fab Fragment Goat Anti-Mouse IgG (H+L) | 115-007-003 | Jackson ImmunoResearch Laboratories |
| ImmPACT AMEC Red Substrate Kit, Peroxidase (HRP) | SK-4285 | Vector Laboratories |
| Mayer Hematoxylin | C0303 | DiaPath |
| Glycerol | G5516 | Sigma-Aldrich |
| Rubber cement | Fixogum | Marabu |

## Table S4. List of antibodies used in immunohistochemical staining

| Marker | Clonality | Host | Clone | Vendor | Cat No. |
|---|---|---|---|---|---|
| NLRP3 | polyclonal | Rabbit | | Novus Biologicals | NBP2-124466 |
| ASC | monoclonal | Rabbit | E1E3I | Cell Signaling Technology | 13833S |
| Caspase-1 | monoclonal | Mouse | 14F468 | Novus Biologicals | NB100-56565 |
| IL-1B | polyclonal | Rabbit | | Abcam | ab9722 |
| IL-18 | monoclonal | Rabbit | EPR19954-188 | Abcam | ab243091 |
| IL-1RA | monoclonal | Rabbit | EPR6483 | Abcam | ab124962 |
| IL-18BP | polyclonal | Rabbit | | Novus Biologicals | NBP2-38481 |
| CK AE1+AE3 | polyclonal | Mouse | AE1/AE3 | Dako Agilent | M3515 |
| MUC1 | monoclonal | Mouse | MRQ-17 | Cell Marque | 290M-16 |
| MUC2 | monoclonal | Mouse | MRQ-18 | Cell Marque | 291M-16 |
| MUC5 | monoclonal | Mouse | CLH2 | Leica Biosystems | NCL-MUC-5AC |
| MUC6 | monoclonal | Mouse | MRQ-20 | Cell Marque | 293M-96 |
| CDX2 | monoclonal | Rabbit | EPR2764Y | Cell Marque | 235R-16 |

## References


1. Akturk G, Sweeney R, Remark R, Merad M, Gnjatic S. Multiplexed Immunohistochemical Consecutive Staining on Single Slide (MICSSS): Multiplexed Chromogenic IHC Assay for High-Dimensional Tissue Analysis. Methods Mol Biol 2020; 2055: 497–519.

2. Tissue expression of NLRP3 - Summary - The Human Protein Atlas. https://www.proteinatlas.org/ENSG00000162711-NLRP3/tissue (accessed 3 May2024).

3. Tissue expression of PYCARD - Summary - The Human Protein Atlas. https://www.proteinatlas.org/ENSG00000103490-PYCARD/tissue (accessed 25 Apr2024).

4. Tissue expression of CASP1 - Summary - The Human Protein Atlas. https://www.proteinatlas.org/ENSG00000137752-CASP1/tissue (accessed 25 Apr2024).

5. Jia Y, Zhao J, Liu M, Li B, Song Y, Li Y et al. Brazilin exerts protective effects against renal ischemia-reperfusion injury by inhibiting the NF-κB signaling pathway. Int J Mol Med 2016; 38: 210–216.

6. Tissue expression of IL1RN - Summary - The Human Protein Atlas. https://www.proteinatlas.org/ENSG00000136689-IL1RN/tissue (accessed 13 May2024).

7. Tissue expression of IL18 - Summary - The Human Protein Atlas. https://www.proteinatlas.org/ENSG00000150782-IL18/tissue (accessed 25 Apr2024).

8. Tissue expression of IL18BP - Summary - The Human Protein Atlas. https://www.proteinatlas.org/ENSG00000137496-IL18BP/tissue (accessed 26 Apr2024).

9. Bankhead P, Loughrey MB, Fernández JA, Dombrowski Y, McArt DG, Dunne PD et al. QuPath: Open source software for digital pathology image analysis. Sci Rep 2017; 7: 1–7.

10. Cardona A. TrakEM2: an ImageJ-based program for morphological data mining and 3d modeling. In: Proc. ImageJ User and Developer Conference. 2006https://www.researchgate.net/profile/Albert-Cardona-2/publication/228421998_TrakEM2_an_ImageJ-based_program_for_morphological_data_mining_and_3d_modeling/links/5475383d0cf29afed6126a73/TrakEM2-an-ImageJ-based-program-for-morphological-data-mining-and-3d-modeling.pdf.

11. Berg S, Kutra D, Kroeger T, Straehle CN, Kausler BX, Haubold C et al. ilastik: interactive machine learning for (bio)image analysis. Nat Methods 2019; 16: 1226–1232.

12. Schindelin J, Arganda-Carreras I, Frise E, Kaynig V, Longair M, Pietzsch T et al. Fiji: an open-source platform for biological-image analysis. Nat Methods 2012; 9: 676–682.

13. Choudhury KR, Yagle KJ, Swanson PE, Krohn KA, Rajendran JG. A robust automated measure of average antibody staining in immunohistochemistry images. J Histochem Cytochem 2010; 58: 95–107.
